# New methods to identify high peak density artifacts in Fourier transform mass spectra and to mitigate their effects on high-throughput metabolomic data analysis

**DOI:** 10.1007/s11306-018-1426-9

**Published:** 2018-09-17

**Authors:** Joshua M. Mitchell, Robert M. Flight, Qing Jun Wang, Richard M. Higashi, Teresa W.-M. Fan, Andrew N. Lane, Hunter N. B. Moseley

**Affiliations:** 1 Department of Molecular & Cellular Biochemistry, University of Kentucky, Lexington, KY USA; 2 Markey Cancer Center, University of Kentucky, Lexington, KY USA; 3 Center for Environment and Systems Biochemistry and the Resource Center for Stable Isotope Resolved Metabolomics, University of Kentucky, Lexington, KY USA; 4 Institute for Biomedical Informatics, University of Kentucky, Lexington, KY USA; 5 Department of Toxicology & Cancer Biology, University of Kentucky, Lexington, KY USA; 6 Department of Ophthalmology and Visual Sciences, University of Kentucky, Lexington, KY USA

**Keywords:** Fourier transform, Mass spectrometry, Artifact, Data analysis, Metabolomics

## Abstract

**Introduction:**

Direct injection Fourier-transform mass spectrometry (FT-MS) allows for the high-throughput and high-resolution detection of thousands of metabolite-associated isotopologues. However, spectral artifacts can generate large numbers of spectral features (peaks) that do not correspond to known compounds. Misassignment of these artifactual features creates interpretive errors and limits our ability to discern the role of representative features within living systems.

**Objectives:**

Our goal is to develop rigorous methods that identify and handle spectral artifacts within the context of high-throughput FT-MS-based metabolomics studies.

**Results:**

We observed three types of artifacts unique to FT-MS that we named high peak density (HPD) sites: fuzzy sites, ringing and partial ringing. While ringing artifacts are well-known, fuzzy sites and partial ringing have not been previously well-characterized in the literature. We developed new computational methods based on comparisons of peak density within a spectrum to identify regions of spectra with fuzzy sites. We used these methods to identify and eliminate fuzzy site artifacts in an example dataset of paired cancer and non-cancer lung tissue samples and evaluated the impact of these artifacts on classification accuracy and robustness.

**Conclusion:**

Our methods robustly identified consistent fuzzy site artifacts in our FT-MS metabolomics spectral data. Without artifact identification and removal, 91.4% classification accuracy was achieved on an example lung cancer dataset; however, these classifiers rely heavily on artifactual features present in fuzzy sites. Proper removal of fuzzy site artifacts produces a more robust classifier based on non-artifactual features, with slightly improved accuracy of 92.4% in our example analysis.

**Electronic supplementary material:**

The online version of this article (10.1007/s11306-018-1426-9) contains supplementary material, which is available to authorized users.

## Introduction

Fourier transform mass spectrometry (FT-MS) provides high performance in terms of sensitivity, resolution, and mass accuracy simultaneously. These combined capabilities provide tangible analytical and interpretative improvements including: (i) the ability to resolve distinct isotopologues with identical unit masses but different accurate masses (Higashi et al. 2014), enabling multi-element isotopic natural abundance correction (Moseley 2010; Carreer et al. 2013); (ii) improved assignment accuracy (Kind and Fiehn 2006); and (iii) the detection of metabolites in the sub-femtomolar range (Eyles and Kaltashov 2004; Dettmer et al. 2007). In the metabolomics field, these improvements permit more complicated, but more informative experimental designs such as the use of multiple isotope-labeled precursors in stable isotope-resolved metabolomics (SIRM) experiments (Yang et al. 2017). Multiple isotope-labeling experiments provide necessary information to elucidate unknown metabolic pathways (Creek et al. 2012; Higashi et al. 2014), quantify relative fluxes through connected metabolic pathways (Hiller et al. 2010), identify multiple pools of a given metabolite in different compartments (Fan et al. 2012), and identify active metabolic pathways under various cellular conditions (Moseley et al. 2011; Sellers et al. 2015; Verdegem et al. 2017). These informational gains enable more complete modeling of cellular metabolism and better understanding of physiological and pathological processes at a mechanistic level, facilitating the identification of potential therapeutic targets (Fan et al. 2009) and the quantification of differential drug response (Harris et al. 2012).

While the advantages of FT-MS are significant, when deployed in a high-throughput environment, the volume of data produced requires automated tools for data reduction, quality control, feature assignment, and downstream analyses. These requirements are amplified when FT-MS is used with direct infusion where existing and well-validated assignment methods that rely on orthogonal sources of information (such as chromatography) are either not applicable or would further increase the amount of data to be processed. Therefore, without automation, rigorous assignment of non-polymeric biomolecules in untargeted MS analyses remains difficult even with FT-MS’s high resolution.

In untargeted FT-MS metabolomics approaches, m/z database-based assignment tools such as LipidSearch (Peake et al. 2013) and PREMISE [in-house tool developed within the Center for Environment and Systems Biochemistry (CESB)] have been used to assign FT-MS features observed in direct infusion experiments (Lorkiewicz et al. 2012; Yang et al. 2017). These database approaches have a low computational overhead and can be tailored to the biological system being studied. However, tailored m/z databases limit discovery—a stated goal of many untargeted analyses, introduce assignment bias (Moseley 2013), and have difficulty disambiguating possible assignments. Additionally, most assignment algorithms assign MS1 spectral features only by comparing observed m/z with database m/z values. This is statistically error-prone due to a lack of aggregate, cross-validating evidence (Kind and Fiehn 2006). Furthermore, all analytical techniques have the potential to generate artifactual signals due to instrumental or data processing limitations. These signals obviously do not represent the underlying biochemistry of a sample, and at best complicate data interpretation and at worst lead to incorrect interpretations. As untargeted experiments become more popular and larger, as in the field of metabolomics (Goodacre et al. 2004), the ability to distinguish sample-specific signals from artifactual signals becomes increasingly desirable as most existing assignment methods assign both artifactual and non-artifactual peaks (Mahieu and Patti 2017).

Artifactual peaks in MS can be divided into two major types. The first type is chemical noise resulting from unintended ions during data acquisition due to, for example, the presence of contaminant compounds (Keller et al. 2008) (e.g. plasticizers and keratin) and spontaneous chemical reactions during analysis [e.g. molecular rearrangements (McLafferty 1959)]. For chemical noise, the artifactual signals represent actual ions that are not directly representative of sample analytes.

The second type of artifactual peaks do not correspond to actual ions. The cause of this type of artifact typically depends upon the mass spectrometry platform on which the spectra are acquired. Fourier transformation of raw time domain data, the free induction decay (FID), is well-known to generate FT-specific artifacts like sinc wiggles in nuclear magnetic resonance (NMR) spectra (Hore 1985), side lobes in FT-infrared spectra (Griffiths and Pariente 1986), and peak ringing in FT-MS. Previous studies of specific artifacts in FT-MS have focused on “harmonic peaks” (Mathur and O’Connor 2009) and “sidebands” or “peak ringing” (Miladinović et al. 2012). In this study, we have mainly developed new methods to rigorously identify fuzzy sites and to mitigate their effects on metabolomic data analyses.

## Materials and methods

### Fuzzy site artifact detection

**Fig. 1:**
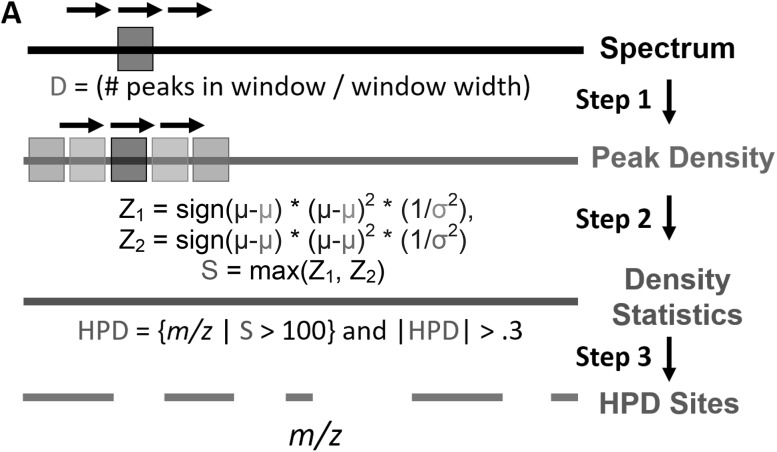
Automated HPD-site detection. The HPD artifact detection algorithm in three steps: first, a peak density metric is calculated for the spectrum using a sliding window method (1 m/z window, 0.1 m/z increment); second, a set of N + 1 windows and the peak density metric are used to calculate a peak density statistic for each portion of the spectrum. This metric flattens out density differences due to signal-to-noise differences or baseline differences and highlights spectra with HPD artifacts (Fig. 3e–h). Filtering this metric reveals the location of the HPD artifacts

We observed three unique artifact types that share the high peak density (HPD) property in otherwise peak-sparse spectra: fuzzy sites, ringing and partial ringing. Since fuzzy sites were present in most of our spectra, we primarily developed a tool for fuzzy site detection based on this HPD property using the Python programming language (Van Rossum and Drake Jr 1995) version 3.4 and Numpy (Walt et al. 2011) for accelerating calculations. Starting with a peaklist in a Javascript Object Notation (JSON) format (Supplemental Fig. S2), the detector first parses and sorts the peaks in ascending order of their m/z values, needed for binary searching of the peaklist. A 1 m/z window (top black box in Fig. 1) is then slid across the spectrum in 0.1 m/z increments. At each increment, two binary searches are used to find all peaks within the window that are then counted to give a peak density metric (Step 1 in Fig. 1) that is assigned to the middle m/z of the window.

High peak density artifacts can be found through the comparison of the actual peak density at a given m/z to the expected peak density derived from surrounding regions (Step 2). In this operation, N pairs of non-overlapping ‘reference’ windows (yellow and purple boxes in the peak density line of Fig. 1) distributed symmetrically around a single ‘test’ window (center black box in the peak density line of Fig. 1) are moved across the spectrum in 0.1 m/z increments. The test window is the spectral region being tested for HPD phenomena using the reference windows as estimates of “expected” peak density. At each increment, the mean and standard deviation of the peak density is calculated for each pair of reference windows, with each reference window 3 m/z in width. The test window is then assigned a density statistic value S (Fig. 1), a chi-squared inspired metric (i.e., the peak density normalized with respect to expected peak density and variance generated from surrounding regions). By taking the maximum value of S, sensitivity is maximized, enabling detection of HPD even if one pair of reference windows contains HPD. Although higher values of N are theoretically superior, testing demonstrated no significant improvement for N > 2. At the ends of the spectra, only the left and right reference windows are used.

In the final step, the continuous subdomains of m/z space at least 0.3 m/z in width (smaller than empirically observed HPD artifacts) and with density statistic values over 100 are reported (Step 3 in Fig. 1). These reported regions very likely contain some form of HPD phenomena as they have significantly higher peak densities as compared to neighboring regions.

### Samples analyzed by FT-MS

Five different sets of samples were used to investigate HPD artifacts on our FT-MS platforms. Sample A are solvent blanks with and without Avanti SPLASH™ Lipidomix® Mass Spec Standard added. Sample B are IC-MS standards prepared from NSG mice livers. Sample C are ethylchloroformate (ECF) solvent standards. Sample D are paired lipid extracts from human non-small cell lung cancer and non-cancer lung tissue samples. These samples are described in greater detail in the supplemental materials.

### FT-MS instruments

Several FT-MS instruments were analyzed to determine the instrument dependence of the HPD artifacts. These instruments include three Thermo Tribrid Fusion instruments (named Fusion 1, 2 and 3), a Thermo Fusion Lumos Tribrid (Lumos), a Thermo Q-Exactive + and a Bruker SolariX instrument. Except for the SolariX, all instruments are orbitraps. Additional instrument details are described in the Supplemental Materials.

## Results

### Manual investigation of artifacts

**Fig. 2:**
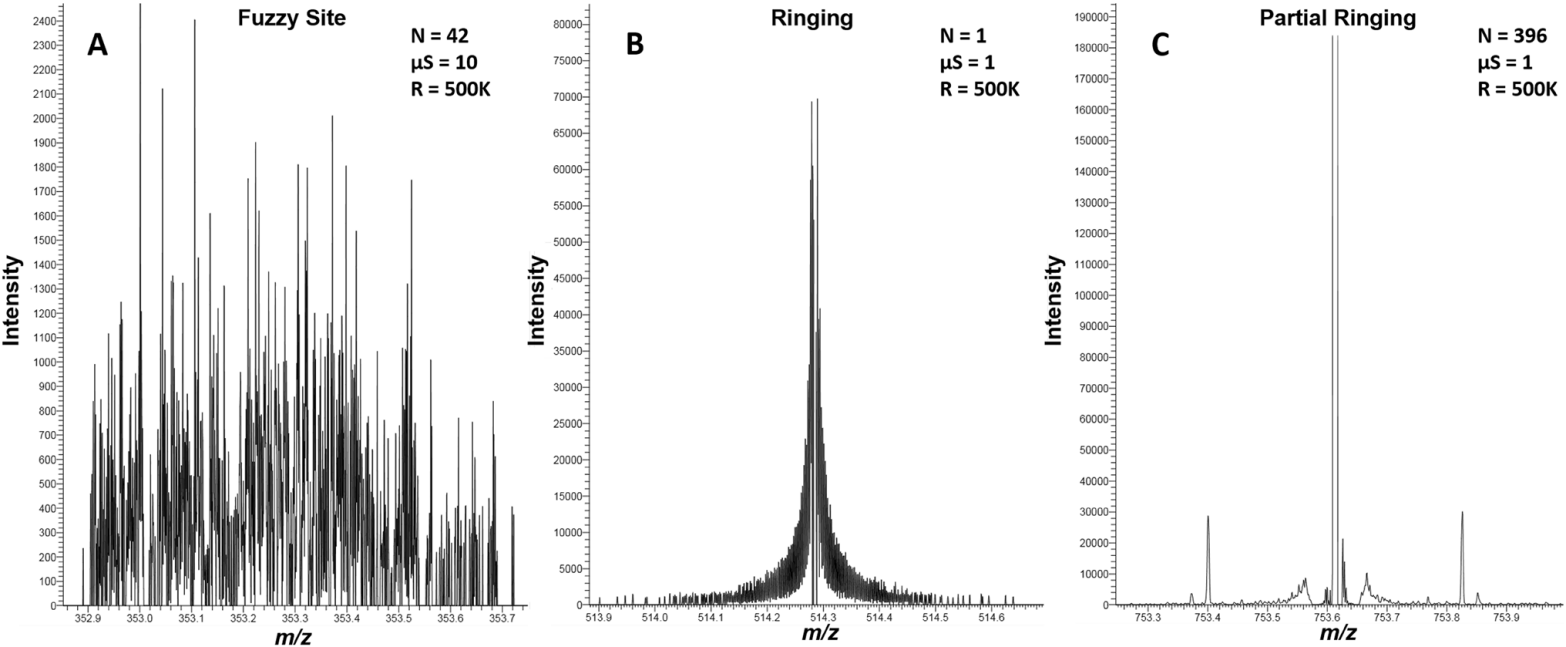
Three types of HPD artifacts. We observed three subclasses of HPD artifacts. The first is the fuzzy site (**a**, sample D), which we believe is a novel artifact type. The second is ringing, a well-known FT-MS artifact where a single intense peak has many side peaks (**b**, sample B). We only observed ringing at the scan level. The third artifact is partial ringing which is a ringing-like artifact at the aggregate level (**c**, sample A). R is the resolution setting used for data acquisition, µS is the microscan setting, and N is the number of scans aggregated to create the spectrum

During FT-MS metabolomics data analysis, we manually inspected several hundred spectra and observed HPD artifacts. Figure 2 illustrates the three major types of HPD artifacts observed: fuzzy sites, peak ringing, and peak partial ringing, with all three appearing distinct at both the aggregate and scan level. Fuzzy sites were present in nearly all spectra from our Fusion instruments, while peak ringing and partial ringing were relatively rare. Although all three artifacts are obvious upon manual inspection with fuzzy sites being far more common than ringing and partial ringing, the high-throughput use of FT-MS-based experiments necessitates automated methods for their efficient identification.

### General HPD detection across FT-MS instruments

**Fig. 3:**
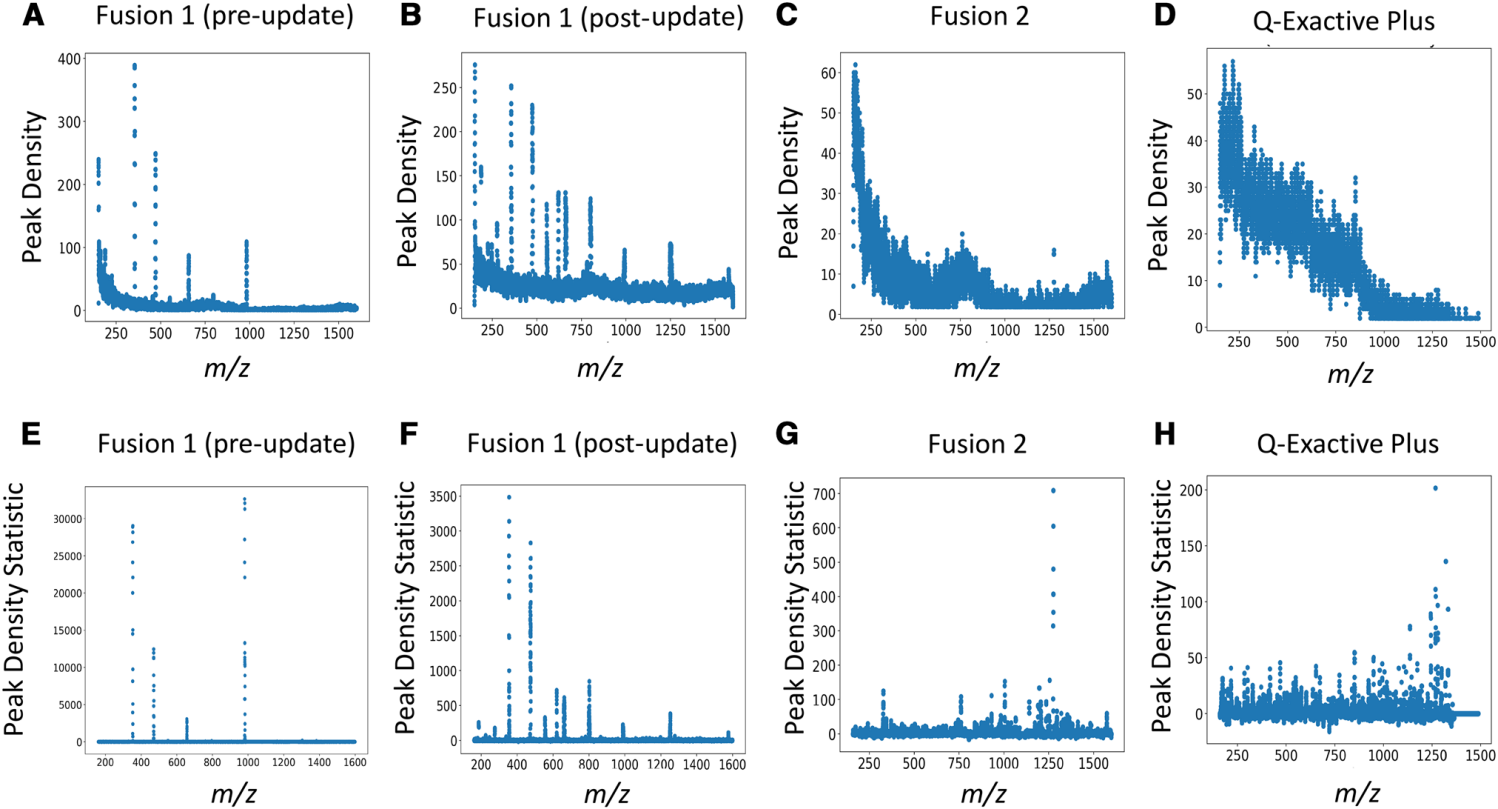
Peak density and peak density statistics. Peak density metric and statistic plots produced by our HPD-detector tool highlight the impact of the instrument on peak density and HPD artifact location. All instruments have higher peak densities at lower m/z, representing trends in signal-to-noise and digitization with respect to m/z in FT-MS. The sharp spikes in peak density correspond to HPD artifacts. The locations of these spikes on Fusion 1 are different before and after the firmware update (**a, b**), suggesting instrument-level data processing is related to HPD generation. **e**–**h** show the effectiveness of our peak density statistic metric for flattening the non-constant baseline observed in plots of the raw peak density. Without this correction, identifying HPD regions reliably is difficult. **a**–**c, e**–**g** were generated from spectra acquired using sample C. **d** and **h** were generated from spectra acquired using sample E

Using the HPD artifact detector, we generated plots of peak density for a variety of example spectra across various FT-MS instruments (Fig. 3). The trends in peak density vary between instruments, samples, and across m/z. In general, peak density decreases with increasing m/z and a monotonic trend was observed for all instruments except the SolariX and Lumos (Fig. 3a–d; Supplemental Fig. S1 upper panels). This observation is partially explained by differences in signal-to-noise and decreasing digitization with increasing m/z due to the nonlinear relationship between the observed frequency and m/z. From these plots, there exists no good cutoff based on raw peak density to identify HPD sites. In contrast, our statistical approach (i.e. peak density statistic) compensates for systematic changes in peak density, revealing regions of significantly higher peak densities with respect to average peak density of neighboring regions (Fig. 3e–h; Supplemental Fig. S1 lower panels).

Although fluctuations in peak densities are expected due to differences in the distribution of compounds in m/z space, this fails to explain the large spot increases (spikes) in the peak density statistic present in the spectra. Similar spikes are observed even in blanks and in scans from failed injections, which contain little analyte and solvent intensity, respectively. Furthermore, analytical samples derived from the same biological sample have HPD regions at different locations with different instruments and these artifact locations differ before and after a firmware update on the same Fusion 1 machine (Fig. 3a, b, e, f). Manual inspection of a subset of the detected HPD regions consistently failed to identify peak patterns that are explainable by chemical phenomena (e.g. isotopologues, different charges, etc.). Together, these findings support an artifactual basis for these HPD regions of spectra and suggest an instrument-level source.

### Detection and characterization of fuzzy sites

**Fig. 4:**
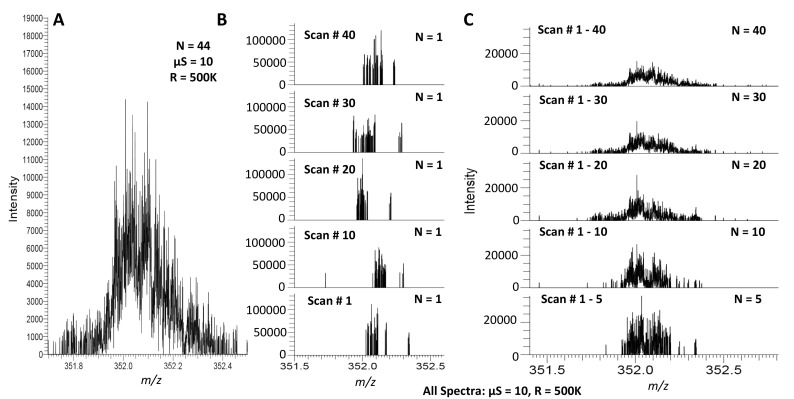
Fuzzy sites at the Aggregate and Scan Level. A typical fuzzy site **a** occupies 0.5–3 m/z at the aggregate level and has a distinct ‘fuzzy’ appearance due to very high peak density (this image is identical to Fig. 2a). At the scan level, only a subdomain of the m/z occupied by the fuzzy site contains peaks; the subdomain with peaks varies from scan-to-scan (**b**). As increasingly more scans are aggregated together, the peak distribution converges to the pattern observed at the aggregate level (**c**). All panels were generated using sample A. R is the resolution setting used for the acquisition, µS is the microscan setting, and N is the number of scans aggregated to create the spectrum

At the aggregate spectrum (sum of scans) level (Figs. 2a, 4a, c), fuzzy sites have HPD characteristics and a Gaussian-like distribution of peak intensities between the noise baseline and presumed signal peaks. The intermediate intensities of these peaks make identifying and filtering these regions by intensity alone difficult. Fuzzy sites, like other HPD artifacts, have peak m/z differences that are not explainable by isotopologue, charge, or harmonic patterns. Fuzzy sites vary in size from 0.5 to up to 3 m/z, with larger fuzzy sites found at higher m/z. Typically, a spectrum with fuzzy sites contains multiple fuzzy sites. Collectively, these sites can represent a significant portion of the total peaks over a much smaller portion of the total m/z range. Fuzzy site location varies between analytical replicates on the same instrument and with sample composition (Supplemental Fig. S7). Fuzzy sites have been observed in samples with a failed or no injection as well.

Fuzzy sites also have interesting properties at the scan-level. While the timing between scans and injection as well as inconsistencies between injections can result in non-perfect scan-to-scan correspondence between peaks (e.g. a peak is present in scan X, but not in scan Y), peaks of the same chemical origin should appear consistently between scans near their true m/z, roughly within the resolution of the instrument. However, at the scan level, the peaks in a mass range identified as a fuzzy site at the aggregate level have very low peak correspondence (Fig. 4b). In any given scan, only sections of the fuzzy site region will have peaks and those sections that are populated with peaks change from scan to scan. However, as increasingly more scans are added together, the Gaussian-like distribution of a fuzzy site at the aggregate level becomes clearer (Fig. 4a, c). Furthermore, resolution does not change the presence of fuzzy sites (Supplemental Fig. S5). Fuzzy sites appear distinct from either peak ringing or partial peak ringing and represent a novel class of artifact not previously described in the FT-MS literature.

Fuzzy sites were first observed on Fusion 1. After developing our methods using Fusion 1 spectra, spectra from other instruments were examined for fuzzy sites to determine if these artifacts were limited to only one instrument. To date, we have observed fuzzy sites in spectra from every non-Lumos Tribrid Fusion instrument examined. However, we did not find fuzzy sites in spectra from other types of FT-MS instruments (Lumos, Q Exactive+, SolariX).

Using our largest dataset (sample D), we were able to evaluate the robustness of our tool with respect to identifying fuzzy sites in spectra from our Fusion 1 and Fusion 2 instruments. For every region reported by our fuzzy site detector tool, we manually inspected 50 spectra to verify if a fuzzy site was present or not. Additionally, the entire spectrum was completely inspected manually for additional fuzzy sites that were not detected by our tool. The detailed results of this analysis are shown in Table S2 and across Fusion 1 and Fusion 2 a sensitivity of 92.1% and a specificity of 33.5% was achieved.

### Fuzzy site locations are biological unit specific, class specific, and instrument specific

As shown in Supplemental Figure S7, fuzzy site location varies between spectra and appears to shift significantly (a shift far greater than the resolution of the instrument) with changes in sample composition. The dependency of fuzzy site location on sample composition is a potential problem for real metabolomics applications of FT-MS, if fuzzy site features are not eliminated from downstream analyses. To demonstrate this, we compared FT-MS spectra of the lipid samples extracted from paired cancer and non-cancer lung tissue slices (sample D). Due to the differences in the concentrations of various metabolites between cancer and non-cancer tissues, we would anticipate that features assigned to the spectra of different sample classes (i.e. cancer and non-cancer) could be used to distinguish these sample classes. However, if fuzzy sites vary with sample class as well, artifactual features will also distinguish sample class without directly reflecting the underlying biochemical differences between these classes.

Using the fuzzy site detector, HPD regions were identified in every spectrum from sample set D. (a) The plot shows that some fuzzy sites are consistent across paired cancer (red) and non-cancer (green) spectra from the same patient. (b) A shifting based on sample class (green vs. red) is observed for some consistent fuzzy sites (specific example regions are shown in Fig. 6). (c) Different consistent fuzzy sites are observed for each instrument, Fusion 1 (black) and Fusion 2 (blue). Across all three plots, the scattering of inconsistent fuzzy sites observed largely at m/z > 1000 represent false positive regions. Spectrum number is an arbitrary index assigned to each spectrum for each plot.

**Fig. 5:**
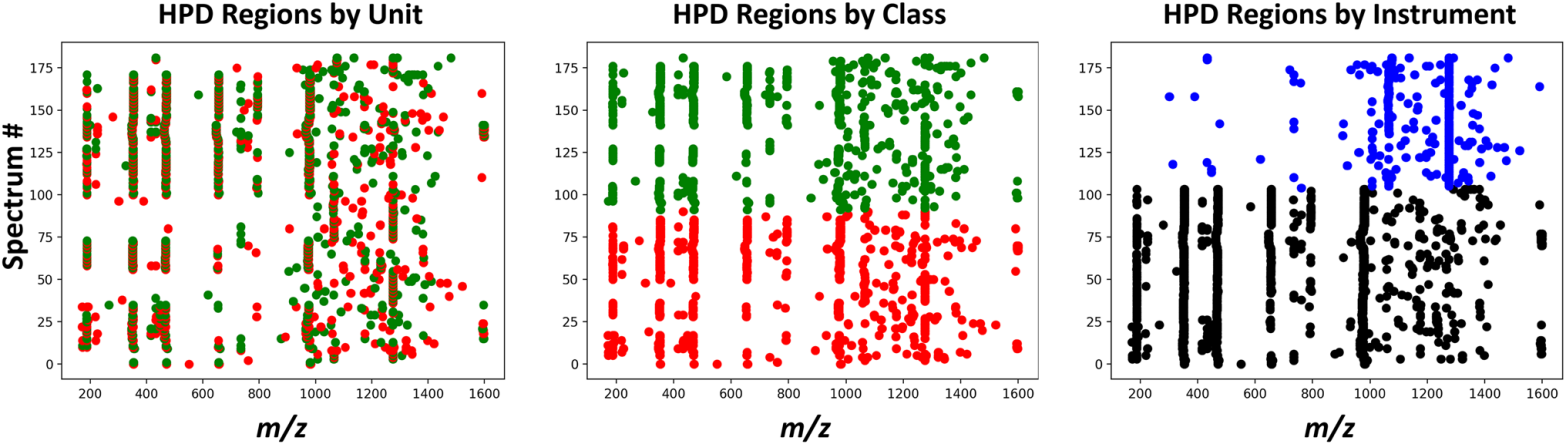
HPD regions depend on biological unit, sample class, and instrument

**Fig. 6:**
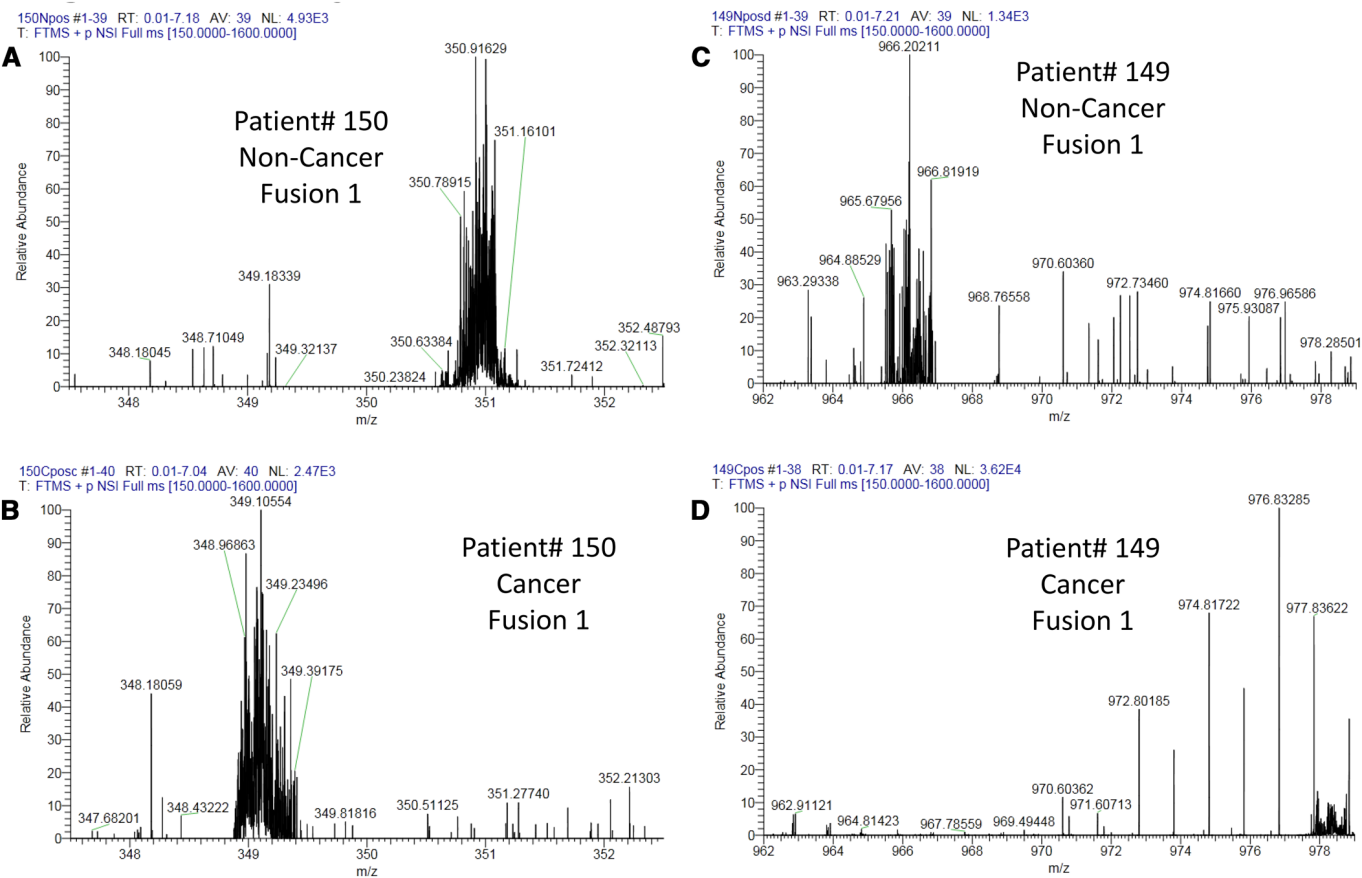
Example fuzzy site locations that vary with sample class. The location of fuzzy sites in spectra from the same biological unit can differ significantly based on sample class (cancer vs. non-cancer). **a** and **b** illustrates one fuzzy site whose location varies by rough 2 m/z between sample class. **c** and **d** shows a single fuzzy site whose location varies by over 12 m/z between sample class

Figure 5 shows the location of the detected HPD regions for every spectrum in sample set D, illustrating a clear relationship between consistent HPD site location and sample origin (biological unit, i.e. patient, Fig. 5a), sample class (Fig. 5b) and instrument (Fig. 5c). HPD regions with less consistency are spurious false positives. Figure 6 shows clear shifts in fuzzy site locations within cancer and non-cancer spectra derived from the same patient. In one example, the fuzzy site appears to have shifted by over 12 m/z units.

This creates a potential confounding factor or batch effect in all downstream statistical analyses if spurious changes in sample conditions introduce new sample-specific artifacts. For example, machine learning methods such as random forest (Breiman 2001) are trained with known classes of samples in order to classify unknown samples into one or more known classes based on spectral features identified in the training set of samples. However, these techniques rely upon an important assumption that detectable artifacts are not confounded with sample class. The large number of sample-specific artifactual peaks produced by fuzzy sites can hijack the classifier training, anchoring the classification to artifacts that may change due to unforeseen sample conditions in unknown samples or unforeseen changes in the analytical instrument.

These hypothetical problems arising from artifact batch effects are clearly demonstrated in Supplemental Table S3. Twenty random forests were trained on LipidSearch assignment features derived from spectra covering a mass range of 150–1600 m/z acquired from non-polar extracts of paired cancer and non-cancer lung tissue samples (sample D). We used only spectra from Fusion 1 to eliminate the instrument as a potential bias and we dropped features present in < 25% of either the cancer or non-cancer classes. Each classifier was built using the R randomForest package (version 4.6-14) from CRAN. Default package options were used except with the number of trees set at 1000. The top features based on mean importance of each feature across the 20 random forests are reported. Consistent HPD regions are identified first by determining the m/z range that contain an HPD site in at least 10% of the samples. Features with an m/z in one of the consistent HPD regions are considered HPD-tainted features and are presumed artifacts. Without HPD feature removal, 6 out of the top 30 features are HPD features (Table S3a). Removing features observed only in a sample’s HPD regions results in a reduction but not complete abolition of HPD-features (Table S3b), since this removal indirectly encodes sample class via the inflation of the importance of features in m/z regions that overlapped with HPD sites in the other class. Only when the consistent HPD regions are removed from every sample does no HPD feature makes it into the top 30 mean importance list (Table S3c), either directly or indirectly. With sample specific and no artifact removal, classification errors of 13.46% and 3.77% are achieved for cancer and non-cancer respectively and consistent HPD removal achieved 11.54% and 3.77%, representing a minor improvement in this example. Confusion matrices for classification are provided (Table S3d–f) The ability to retain classification accuracy with artifact removal implies that we are not significantly removing any important information through our artifact removal process, while increasing the likelihood that the classification is based on true biological variance. The similarity in the important features between the important non-HPD features across all three removal methods supports this hypothesis.

## Discussion

### Origin of FT-MS artifacts

The scan and aggregate level properties of HPD sites strongly support an artifactual origin for HPD features; however, their exact origin has not been confirmed.

Fuzzy sites appear to be evenly spaced in the frequency domain, which is consistent with background radio frequency interference (RFI). RFI artifacts are not unexpected given the sensitivity of the detectors used in FT-MS instruments, but the correlation between fuzzy site location and sample class is surprising. The change in fuzzy site location after a firmware update and the presence of fuzzy sites in every Fusion instrument we have tested could indicate an internal source of the RFI. An internal source could produce class-specific fuzzy sites if the state of the components producing the RFI changes in some manner with sample composition. However, a more likely hypothesis is that the source of the RFI is the nanoelectrospray system (or other external device that is part of the deployed FT-MS system). The nanoelectrospray system is in direct contact with the sample and operates at high voltage as part of its normal operation. Any interaction between the voltage of the electrospray system, the pulses used to ionize the sample and the sample itself could result in the generation of RFI signals that result in sample-specific fuzzy sites. Unfortunately, robustly investigating these hypotheses is difficult and ultimately not relevant to fixing these artifacts in already acquired data. Discussions with Thermo staff support an RFI origin for fuzzy sites, although the source of the interference remains unclear.

The origin of partial ringing is even less clear. Since partial ringing does not occur in most spectra and across multiple instruments, if it is an RFI-based phenomenon, the source of that RFI is likely transient and external. Due to its rarity and with no obvious relationship to sample class, partial ringing is less of a burden to data analysis. Likewise, rarity of ringing largely mitigates its harmful effect on data analysis. Although ringing can be caused by truncated FIDs, ringing on the Fusion is due to insufficient digitization of the FIDs (personal communication with Mike Senko, Thermo Fisher Scientific).

Eliminating the source of these artifacts, particularly fuzzy sites, is challenging. First, the radio environment that an instrument observes constantly changes and is often beyond the control of the instrument’s operator. Second, these artifacts may not be recognized until after spectral acquisition and the original samples are no longer available due to either consumption or degradation. In this case, the only option is to clean up the acquired data before downstream analyses. Third, access to the raw FID data is not always available for a given instrument. In this case, the only option is to remove the artifact peaks in the m/z domain.

LC–MS and other approaches for gathering orthogonal information about features observed in MS offer an approach to mitigate the effects of these artifacts, but are obviously not applicable to direct injection FT-MS experiments. Retention times combined with m/z can often unambiguously identify metabolites where m/z cannot and artifactual features that are ambiguous by m/z alone will lack supporting chromatographic information. As a result, LC–MS is less vulnerable to the artifacts, but is not always suitable for the same set of experiments that direct injection FT-MS can be applied to.

### Mitigating the effects of fuzzy sites on downstream data analyses

As shown in Table S3, HPD artifacts, namely fuzzy sites, impact data analysis. The resulting classifiers can effectively predict sample class; however, these classifiers make extensive use of peaks present in fuzzy sites and thus have no direct molecular interpretation. Although LipidSearch can use orthogonal information (chromatographic retention time, MS2), which presumably could prevent assignment to artifactual peaks, this information is only available for a small fraction of peaks (typically < 10%) in the direct-infusion FT-MS spectra collected.

Building robust classifiers based on true biological variance therefore requires eliminating fuzzy site assignments from the feature list prior to classifier training to mitigate any fuzzy site/sample class confounds. Removing consistent HPD regions from all spectra safely removes artifactual features efficiently and consistently. Additionally, a check on the consistency of the HPD sites minimizes the impact of false positives returned by our tool and effectively increases the sensitivity of our method. Since we could not find a fuzzy site that was consistently missed by our detector, the bagging process of random forest makes the method very robust against artifactual that do not show a clear batch effect.

In this example, regardless of HPD removal, good disambiguation of cancer and non-cancer was achieved (Table S3a–f); however, HPD removal is crucial for ensuring that important features represent true biological variance between sample class. Furthermore, when artifacts and other systematic errors are present, classification accuracy is not necessarily a complete indication of classifier quality or robustness.

Although the removal of consistent fuzzy site regions is efficient, safe and consistent, it does have shortcomings. First, it will remove larger amounts of spectrum as the number of samples and classes increases. Second, when fuzzy site location consistently overlaps with non-artifactual peaks, these peaks will be discarded even if they are not artifactual. Without a per-peak metric for determining if a peak is a likely artifact or not, this is an unavoidable but undesired side effect. Third, not all consistent HPD regions are fuzzy sites. We have observed consistent HPD regions that do not contain obvious fuzzy sites, at least not in the spectra we manually inspected. These regions do contain high numbers of peaks that are too consistent across spectra, have too many peaks to be random noise, and have poor scan-to-scan consistency. Examples of these regions are illustrated in Supplemental Fig. S10. We hypothesize that these sites do contain an HPD artifact of some sort, but thresholding at the instrument level has removed all but the most intense of these artifactual peaks.

## Conclusions

With our fuzzy site and ringing detection methods, we have identified and characterized three distinct types of artifacts that produce large numbers of peaks (up to 1/3 of the peaks in a spectrum): fuzzy sites, ringing and partial ringing.

All three artifacts complicate assignment and confound experimental interpretation; however, our study focuses primarily on fuzzy sites and their detection as they are novel artifacts and are particularly problematic for classification studies. Fuzzy sites are likely the result of RFI and their location correlates with sample class, increasing the probability of class-specific misassignment and the introduction of sample-specific artifactual features. Ultimately, the presence of these artifacts produces brittle classifiers and complicates the characterization of true biological variance between sample classes using direct-injection FT-MS. The results presented in this study reflect both the severity of this artifact for untargeted metabolomics experiments, but also the deficiency in assignment tools specifically designed for untargeted analyses. The methods and tools presented in this study detect and remove fuzzy sites in a non-encoding manner from direct-injection FT-MS spectra (i.e. without encoding sample class as absence of peaks), while providing sufficient protection from fuzzy site artifacts with existing assignment methods. Ultimately, better assignment pipelines will be necessary as experiments grow in scale. A spectral analysis approach that can leverage peak correspondence between scans to infer if a peak is artifactual or real would produce higher quality peaklists for downstream assignment, data analysis, and interpretation.

## Electronic supplementary material

Below is the link to the electronic supplementary material.

Supplementary material 1 (DOCX 2250 KB)

## Data Availability

Code and data used for this manuscript are available here: https://figshare.com/s/700ea5fde9c2229c1f9c.
