# Supplementary material for: New methods to identify high peak density artifacts in Fourier transform mass spectra and to mitigate their effects on high-throughput metabolomic data analysis
Source: Metabolomics. 2018 Sep 17;14(10):125. doi: 10.1007/s11306-018-1426-9 (PMC6153687; doi:10.1007/s11306-018-1426-9)
Supplement: Supplementary file 1 — Supplementary material 1 (DOCX 2250 KB) [file 11306_2018_1426_MOESM1_ESM.docx]

**Supporting Information for**

**New Methods to Identify High Peak Density Artifacts in Fourier Transform Mass Spectra and to Mitigate Their Effects on High-Throughput Metabolomic Data Analysis**

Joshua M. Mitchell^1,2,3^, Robert M. Flight^2,3^, Qing Jun Wang^2,6^, Richard M Higashi^2,3,5^, Teresa W-M Fan^2,3,5^, Andrew N. Lane^2,3,5^, Hunter N.B. Moseley^1,2,3,4,*^

^1^Department of Molecular & Cellular Biochemistry

^2^Markey Cancer Center

^3^Center for Environment and Systems Biochemistry and the Resource Center for Stable Isotope Resolved Metabolomics

^4^Institute for Biomedical Informatics

^5^Department of Toxicology & Cancer Biology

^6^Department of Ophthalmology and Visual Sciences

University of Kentucky, Lexington KY, United States

*Corresponding Author: [hunter.moseley@uky.edu](mailto:hunter.moseley@uky.edu)

**Keywords:** Fourier transform, mass spectrometry, artifact, data analysis, metabolomics

**Abstract:**

The pages contain supporting information, including an additional method specifically developed for detecting ringing and partial ringing artifacts, detailed information regarding the samples analyzed by FT-MS, the instruments utilized in this study, further characterization of fuzzy sites and ringing, eight supplemental figures, and one supplemental table.

**Table of Contents**

**Content Page**

Cover Page S-1

Abstract and Table of Contents S-2

Ringing detector S-3
Samples analyzed by FT-MS S-5

FTMS Instruments Studied S-10

Fuzzy site characteristics varies with resolution and microscan settings S-11

Peak Ringing and Partial Ringing S-12

Figure S-1: Additional Peak Density Examples S-13

Figure S-2: Peaklist JSON S-15

Figure S-3: Contaminants JSON S-16

Figure S-4: Adducts JSON S-17

Figure S-5: Effect of Resolution and Microscan (µS) on Fuzzy Sites S-18

Figure S-6: High μS Fuzzy Site S-19

Figure S-7: Fuzzy Sites vary with Sample Composition S-20

Figure S-8: Ringing Artifacts S-21

Figure S-9: Partial Ringing Artifacts S-22

Figure S-10: Example False Positive Fuzzy Sites S-23

Table S-1: Spectral Acquisition Times for Sample D S-24

Table S-2 Detailed Analysis of Potential Fuzzy Sites S-29

Table S-3 Classification Results and Confusion Matrices S-31

References S-35

**Ringing detection.**

Ringing and partially ringing peaks will present as side bands to primary peaks that are shifted by a small amount in *m/z*. Therefore, the ringing peaks of two different primary peaks are likely to have an m/z difference close to the m/z difference between the two primary peaks. This property can be detected to identify peaks that are likely ringing peaks of a primary peak. The number of m/z matches necessary to infer that a peak is a ringing peak can be determined by finding the mean number of matches in peaks likely to be noise.

Using this approach, we created a dedicated ringing detector in Python 3.4 that identifies sets of ringing peaks for a given real peak based on matching *m/z* differences to expected compounds. The ringing detector requires as input both a peak list and a list of expected *m/z* values (Supplemental Figure 3), each in a JavaScript Object Notation (JSON) format, and a description of the adducts to consider for each ion (Supplemental Figure 4). The default list of expected contaminant *m/z* values was built using the MaConDa database (Weber *et al.*, 2012) of common mass spectrometry contaminants, where each entry represents the non-adducted monoisotopic form of a contaminant, while the adduct and isotopologue file was created manually.

In the first step of the analysis, the ringing detector expands the list of expected *m/z* values by generating their adducts and a set of expected isotopologues as specified in the adduct and isotopologues file. This expansion ensures generation of the most likely isotopologues of each adducted form of each contaminant in the provided contaminant list. With the expanded set of expected *m/z* values calculated, the peaklist is parsed and the peaks are sorted in ascending *m/z* order to enable binary searching by *m/z* value.

Next, a sample of peaks that are from the “noise” is used to estimate the number of m/z matches that will occur at random. First, an estimate of the noise intensity level is calculated as follows. From an ascending list of peak intensities, the ringing detector first identifies the first quartile of the intensity values, i.e. the lowest quartile of intensities, and copies them to a noise intensity list. While the list of intensities is predominantly intensities from noise peaks, we expect the standard deviation of the intensities to be roughly the same as the mean of the intensities, since they are randomly distributed. Therefore, while the standard deviation of the noise intensity list is less than 1.2 times the mean of the noise intensity list, the next 100 intensities are copied to the noise intensity list. At that point, when the standard deviation is larger than the mean of the intensity list significantly, we have an estimate of the noise. Once this process is completed, the median of the noise intensities is calculated. To best separate noise from signal, five times the median noise intensity is considered the intensity cutoff for the spectrum and intensities above the cutoff are very likely “true” signal. A noise sample is then generated by selecting a random set of peaks below the noise cutoff equal in size to the number of peaks above the cutoff.

Using the noise sample, the number of m/z matches at random can be estimated. The ringing detector uses a binary search to find all peaks within +/- 0.05 *m/z* of each *m/z* value in the set of expanded *m/z* values. The *m/z* difference between each found peak and all other peaks in the noise sample (peak mass difference vector) is calculated, as is the *m/z* difference between the expected *m/z* and all other expected *m/z* values (query mass difference vector). The number of matching *m/z* differences within a match-difference *m/z* tolerance between the peak mass difference vector and the query mass difference vector are counted. For the ultra-high resolution FT-MS spectra analyzed in this study, we used a tight match-difference tolerance of ±0.00005 *m/z*. The peak with the most matching *m/z* differences and the number of matches are reported. Since these are noise peaks, these matches are unlikely to be correct and the number of *m/z* difference matches is due to random chance. The mean number of m/z matches plus three times the standard deviation of the distribution of m/z matches is calculated as a cutoff. A peak not in the noise must have more m/z matches than the cutoff to be marked as a ringing peak.

Finally, the ringing detector repeats the search procedure using the entire peaklist. During this search, only peaks with many matching *m/z* differences greater than the match cutoff calculated from the noise peaks are reported as assignments. When ringing or partial ringing occurs, multiple assignments occur for an expected *m/z* value as there are many peaks close to the primary peak that have similar *m/z* differences to other expected compounds.

**Samples analyzed by FT-MS.**

*Sample A: Solvent Blanks with and without Avanti Lipid Standards*

The solvent blank was composed of Isopropanol:Methanol:Chloroform 800 μl:344 μl:200 μl. The solvent blank was mixed with 28 μl 1 M ammonium formate (final ~20 mM; Aldrich #516961), and without or with 70 μl 1:10 diluted Avanti SPLASH™ Lipidomix® Mass Spec Standard (cat# 330707) in MeOH. The solvent blank without or with lipid standards was loaded onto a 96-well polypropylene PCR plate (USA Scientific cat# 1402-9800) and 15 μl was injected into Fusion 1 by direct infusion through an Advion nanomate. Various resolution and microscan settings were tested in positive mode with 7 min acquisition, normal mass range between 150-1600 *m/z*, S-lens RF level 60%, AGC target 1e5, maximum injection time 100 ms, and Easy-IC on.

*Sample B: Mouse Liver Ion Chromatography (IC)MS Standard*

The NOD/SCID gamma (NSG) mouse colony was maintained by the Division of Laboratory Animal Resources at the University of Kentucky. The initial breeding pairs were purchased from The Jackson Laboratory in Bar Harbor, ME. The mice were housed in a climate-controlled environment with a 1410 hours light / dark cycle and lights-on at 0600 hours. The mice had free access to food and water. The mice were feed a liquid diet base containing casein, L-cystine, soy oil, cellulose, mineral mix (AIN-93G-MX), calcium phosphate, vitamin mix (AIN-93-VX), choline bitartrate, tert-butylhydroquinone and xanthan gum purchased from Harlan Laboratories (Madison WI).

Mice were euthanized by spinal dislocation and livers excised and flash frozen in liquid nitrogen within 5 minutes of euthanization. Frozen tissues were ground into powder under liquid nitrogen to <10 μm particles using a Spex freezer mill. Approximately 0.5 g of the powder was extracted with 50 ml acetonitrile:water (6:4, v/v). After centrifugation at 22 kg and 4°C for 20 min, the supernatant containing polar extracts was distributed into aliquots and lyophilized for long-term storage at -80 °C. Immediately before injection, the lyophilized powder was reconstituted with water and 10 μl was injected onto an ICS5000+ system (Dionex) interfaced to the FT-MS (Fusion 2). Data were acquired in negative mode at a resolving power of 500,000 (at *m/z*=200) over 52 min of chromatography. The mass range was set between *m/z* 80 and 700, maximum injection time was 100 ms with 1 microscan, AGC target was 2e5, S-lens RF level was 60%, and Easy-IC was turned on for internal mass calibration. The chromatograph was outfitted with a DionexIonPac AG11-HC-4μm RFIC&HPIC guard (2x50mm) guard column upstream of a DionexIonPack AS11-HC-4μM RFIC HPIC (2x250mm) column (Sun *et al.*, 2017).

*Sample C: ECF Solvent Standard*

The ECF solvent blank was composed of acetonitrile:water 9:1 (v/v) with a concentration of 20 μM NaCl to convert positively charged ions into sodium adducts (Yang *et al.*, 2017).

*Sample D: Paired Human NSCLC Cancer and Non-Cancer Tissue Samples*

Eighty-six patients with suspected resectable stage I or IIa primary non-small cell lung cancer (NSCLC) and without diagnosed diabetes were recruited based on their surgical eligibility. The extent of resection was determined by the surgeon in accordance with clinical criteria. Many of the specimens were obtained from wedge resections which minimizes surgery time while the other specimens were acquired in less than 5 minutes after the pulmonary vein was clamped. Both techniques minimize ischemia in the resected tissues. Immediately after resection, the tumor was transected and section of cancerous tissue and surrounding non-cancer tissue at least 5 cm away from the tumor were immediately flash frozen in liquid nitrogen and stored at <80°C. On-site pathologists confirmed the diagnosis and cancer-free margins on parallel tissue samples. All samples were collected under a University of Louisville approved Internal Review Board (IRB) protocol and written informed consent was obtained from all subjects prior to inclusion in the study (Sellers *et al.*, 2015).

The frozen samples were pulverized under liquid nitrogen to <10 μm particles using a Spex freezer mill, and extracted using a modified Folch method as previously described (Ren *et al.*, 2014). The lipid fraction was supplemented with 1 mM butylated hydroxytolune and then dried by vacuum centrifugation at room temperature. Samples for FT-MS analysis were redissolved 200-500 μl chloroform/methanol (2:1) supplemented with 1 mM butylated hydroxytolune. Reconsitituted lipids samples were diluted in in isopropanol/methanol/chloroform 4/2/1 (v/v/v) with 20 mM ammonium formate (95 μl of solvent for 5 μl of sample) before direct infusion.

*Mass Spectrometry Analysis of Samples C and D*

Ultrahigh resolution (UHR) mass spectrometry was carried out on a Thermo Orbitrap Fusion interfaced to an Advion Nanomate nanoelectrospray source using the Advion “type A” chip, also from Advion, inc. (chip p/n HD_A_384). The nanospray conditions on the Advion Nanomate were as follows: sample volume in wells in 96 well plate – 50 µl, sample volume taken up by tip for analysis – 15 µl, delivery time – 16 minutes, gas pressure – 0.4 psi, voltage applied – 1.5 kV, polarity – positive, pre-piercing depth – 10 mm. The Orbitrap Fusion Mass Spectrometer method duration was 15 minutes, and the MS conditions during the first 7 minutes were as follows: scan type – MS, detector type – Orbitrap, resolution – 450,000, lock mass with internal calibrant turned on, scan range (*m/z*) – 150-1600, S-Lens RF Level (%) – 60, AGC Target – 1e5, maximum injection time (ms) – 100, microscans – 10, data type – profile, polarity – positive. For the next 8 minutes, the conditions were as follows for the MS/MS analysis: MS properties: detector type – Orbitrap, resolution – 120,000, scan range (*m/z*) – 150-1600, AGC Target – 2e5, maximum injection time (ms) – 100, microscans – 2, data type – profile, polarity – negative; monoisotopic precursor selection – applied, top 500 most intense peaks evaluated with minimum intensity of 5e3 counts; data dependent MS^n^ scan properties: MS^n^ level – 2, isolation mode – quadrupole, isolation window (*m/z*) – 1, activation type – HCD, HCD collision energy (%) – 25, collision gas – Nitrogen, detector – Orbitrap, scan range mode – auto *m/z* normal, Orbitrap resolution – 120,000, first mass (*m/z*) – 120, maximum injection time (ms) – 500, AGC target – 5e4, data type – profile, polarity – positive. The ion transfer tube temperature was 275^o^C. (Yang *et al.*, 2017).

*Sample E: Human Plasma*

Mixed gender, unfiltered pooled lithium heparin treated plasma (Seralab Catalog# HMPLLIHP, Lot# BRH1049783) from healthy donors was extracted using previously published protocols (Acharjee *et al.*, 2017). Briefly, 15 µl of plasma was extracted with 100 µl of ultra-pure H_2_O in a glass vial (2 ml). 250 µl of MeOH was added, and lipids were partitioned into 500 µl of methyl-tertiary-butyl ether. Following centrifugation (13,000 rpm, 4°C, 4 min), a 20 µl aliquot of the organic layer was transferred to a 96-well glass coated plate (Thermo Fisher).  95 µl of a solution containing 7.5 mM ammonium acetate in Isopropanol:Methanol (2:1 v/v) was also added to the well.  Direct infusion high-resolution mass spectrometry was performed using on a Q-Exactive+ Orbitrap (Thermo), equipped with a Triversa Nanomate (Advion).  The Nanomate infusion mandrel was used to pierce the seal of each well before analysis, after which, with a fresh tip, 5 μl of sample was aspirated, followed by a 1.5 μl air gap.

**FT-MS Instruments**

To determine the instrument dependence of various artifacts described here, we investigated the spectra from several FT-MS instruments. The first set of instruments includes three Thermo Tribrid Fusion instruments. Fusion 1 (Serial # FSN10115) and Fusion 2 (Serial # FSN10352) are maintained by the Center for Environmental and Systems Biochemistry (CESB) at the University of Kentucky, while Fusion 3 (Serial # FSN 10144) is maintained by the National Resource for the Mass Spectrometric Analysis of Biological Macromolecule at the Rockefeller University. Fusion 1 was delivered to CESB in October 2013. In March of 2016, Fusion 1 had its firmware upgraded to the most recent version at that time. Pre-firmware upgrade Fusion 1 (Fusion 1 – Before) has different HPD artifact patterns than post-firmware Fusion 1 (Fusion 1 – After). Fusion 2 was delivered to CESB in May 2015 with the upgraded firmware. Spectra from a Thermo Scientific Orbitrap Fusion Lumos Tribrid mass spectrometer (Serial # FSN20208, delivery data July 2016), which is maintained by the Proteomics Resource Center at the New York University Langone Medical Center, were also examined. The Lumos represents an improvement upon the original version of the Tribrid Fusion, offering higher resolution and dynamic range. In addition to the Fusion instruments, we also examined spectra from a Thermo Q-Exactive+ instrument, which is another Orbitrap instrument maintained by the High Resolution Metabolomics Laboratory (HRML) at the Institute of Biological, Environmental and Rural Sciences at the Aberystwyth University in the United Kingdom. Also, spectra from a Bruker Solarix instrument (Serial # 150506 A), an ICR-type FT-MS delivered to CESB in April 2014, were examined.

**Fuzzy site characteristics varies with resolution and microscan settings.**

With fuzzy sites found in non-Lumos Tribrid Fusion spectra, we began investigating the effect of two instrument parameters on the appearance of these artifacts. The first parameter examined was resolution, which was a parameter that was improved by the firmware update that changed the HPD properties of Fusion 1. The most up-to-date Fusion has a maximum resolution of 500K at 200 *m/z* (450K before update) and the second parameter was the number of microscans per scan. The number of microscans is the number of FIDs acquired and summed to create the FID that is transformed to produce the scan-level spectrum. Due to the high scan-level variability of fuzzy sites, these settings were of interest as it directly impacts how scan-level FIDs are acquired and processed.
 Using the same sample of solvent blank with lipid standards, spectra were acquired on the Fusion 1 instrument at 3 resolutions (120 K, 240 K and 500 K at *m/z*=200) and 4 microscan settings (1,2,5, and 10 microscans, but only 1,5, and 10 microscans are shown in Supplemental Figure S-5). No combination of settings eliminated the fuzzy sites, but they do change the general appearance of the fuzzy sites. Higher resolution increased the peak density of fuzzy sites, indicating that the peaks within these regions may be sharper (smaller peak widths) than what is indicated at the highest resolution. Higher microscan settings increased the variability in peak intensities. Also, extremely high microscan settings (e.g., µS **=** 350) resulted in broad uniform regions for these fuzzy sites (Supplemental Figure S-6).

**Peak Ringing and Partial Ringing**

Peak ringing is an HPD artifact characterized by the presence of many peaks symmetrically centered around a very intense primary peak. The intensity of these side peaks decreases with increasing distance from the primary peak and the *m/z* interval between each side peak is consistent across the entire artifact. This pattern is clear in Figures 2B and Supplemental Figure S-8A. Peak ringing is an all-or-none phenomenon at the scan level (Supplemental Figure S-8A) and peaks that ring in one scan may not ring in all scans (Supplemental Figure S-8B) and may exhibit other artifact types such as partial ringing (Supplemental Figure S-8C, D). We only observed ringing for peaks with high relative intensity. Peak ringing is a well-known artifact type in FT-based instruments and can be caused by Fourier transformation of a truncated FID (Wood and Mark Henkelman, 1985) or by insufficient digitization of an acquired FID. There are well-known solutions to suppressing these artefacts using processing in the time domain (Guan and Marshall, 1997). These artifacts are well-described in the literature and are summarized here for comparison to partial ringing.

Like ringing, partial ringing occurs around intense peaks (*i.e.*, primary peaks) (Figure S-9A), with the artifactual peaks varying significantly at the scan-level (Figure S-9B-D). At the aggregate level, the artifactual peaks appear symmetrically centered around the primary peak (Figure S-9A), occupying several tenths of an *m/z*, but rarely occurring in the immediate vicinity of the primary peak (Figure S-9B, C). Side peaks from partial ringing are of lower intensity than the primary peak, but their intensities do not necessarily decrease with increasing distance from the primary peak. Although the symmetry of the peaks and the intensity pattern is less apparent in some scans (Figure S-9D), when sufficient scans are aggregated, partial ringing appears smoother and more akin to true ringing except near the primary peak (Figure S-9A).

**Figure S-1: Additional Peak Density Examples.**

We have performed peak density analyses on both our Solarix ICR instrument and a Thermo Lumos Tribrid Fusion instrument, a more advanced version of the Tribrid Fusion. In the ICR, severe HPD phenomena are present due to ringing phenomena. It is not clear if the ringing artifacts from ICR are identical in origin to those in Orbitrap spectra. Our Lumos examples show no obvious HPD artifacts of any kind. In both the ICR and the Lumos, the peak density decreases with increasing peak density but not monotonically.


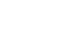

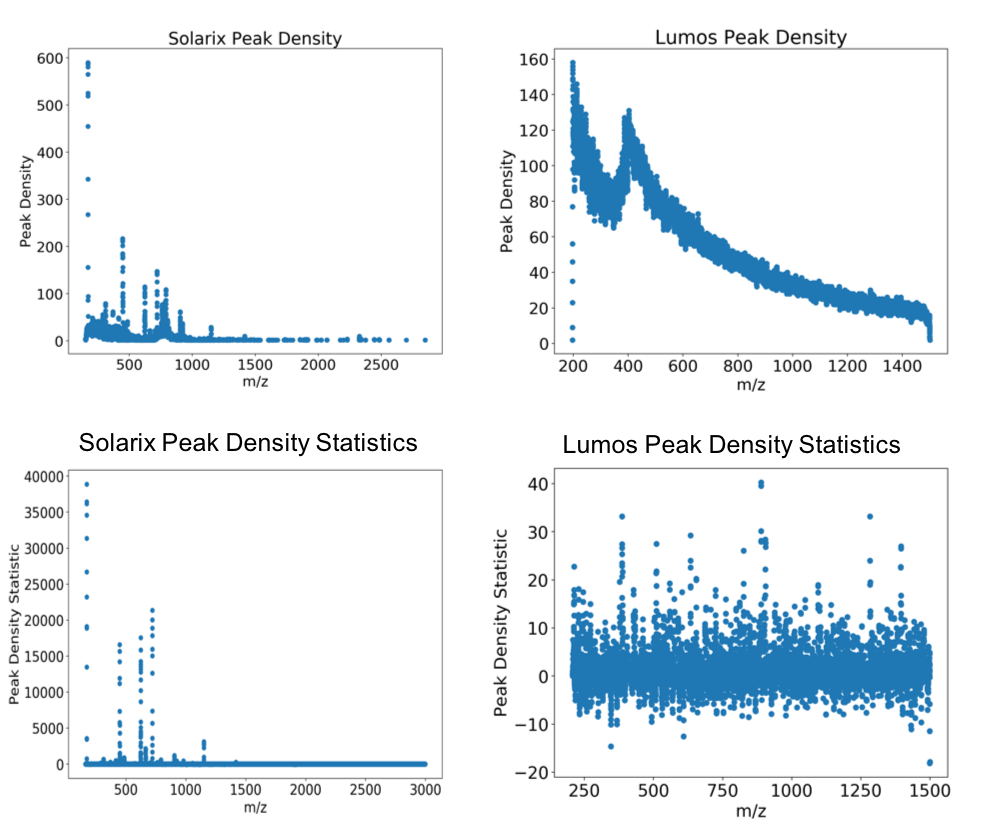


**Figure S-2: Peaklist JSON.**

**{**

**“Peaks”: [**

**“ObservedMZ”: 141.421,**

**“Intensity”: 314.159**

**},**

**{**

**“ObservedMZ”: 161.803,**

**“Intensity”: 54.7356,**

**} …**

**]**

**}**

**Figure S-3: Contaminants JSON.**

**{**

**“Contaminants”: [**

**{**

**“Formula”: “C18H34O4”,**

**“Name”: “Dibutyl Sebacate”,**

**“Mass”: “314.245697”,**

**“Class”: “Plasticiser”,**

**},**

**{**

**“Formula”: “C8H18O3”,**

**“Name”: “Dibutyl carbitol”,**

**“Mass”: “162.125595”,**

**“Class”: “Scintillation cocktail”,**

**}, …**

**]**

**}**

**Figure S-4: Adducts JSON.**

**{**

**“Components”: {**

**“[M+1H1-e_POS]”: {**

**“Max”: 1,**

**“ExtraGroups”: [“Adducts”],**

**“Mass”: 1.007276452191,**

**}**

**}**

**Figure S-5: Effect of Resolution and Microscan (µS) on Fuzzy Sites.** Permuting over multiple resolution and µS settings shows that no combination of tested settings eliminated fuzzy sites, but these settings do change their appearance (A-I). Number of scans collected were set so that total acquisition time was constant (7 minutes). Higher µS increases intensity variance with minimal impact on peak density. Increasing resolution increases peak density but has a lesser impact on peak intensity variance. All panels were generated using Sample A on Fusion 1. **R** is the resolution setting used for the acquisition, **µS** is the microscan setting, and **N** is the number of scans aggregated to create the spectrum.


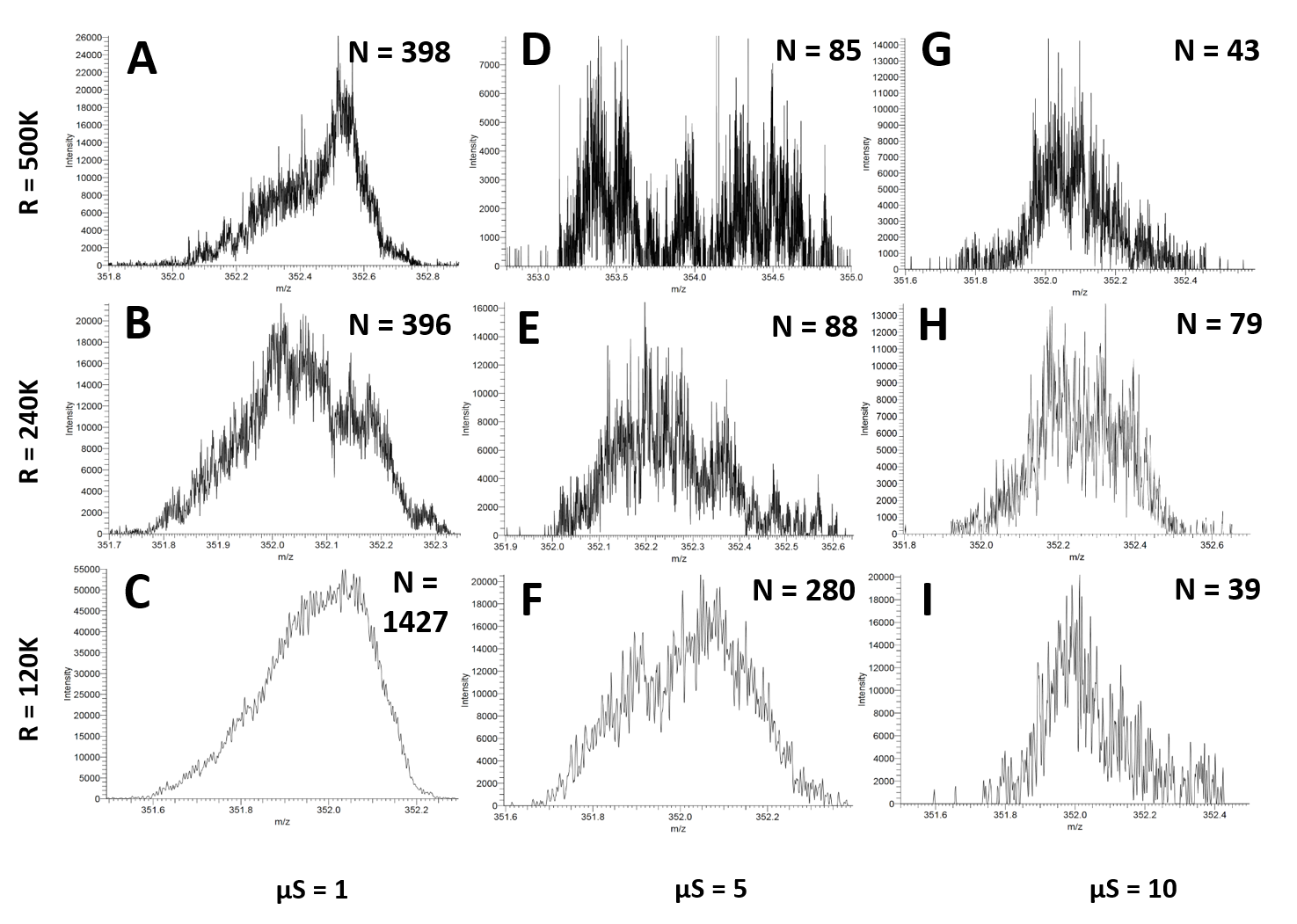


**Figure S-6: High μS Fuzzy Site.**

At very high microscan settings (Sample A), fuzzy sites become almost uniform and span a wide *m/z* window. Although still responsible for artifactual peaks, the significant intensity difference allows the distinguishing of true signal. **R** is the resolution setting used for the acquisition, **µS** is the microscan setting.


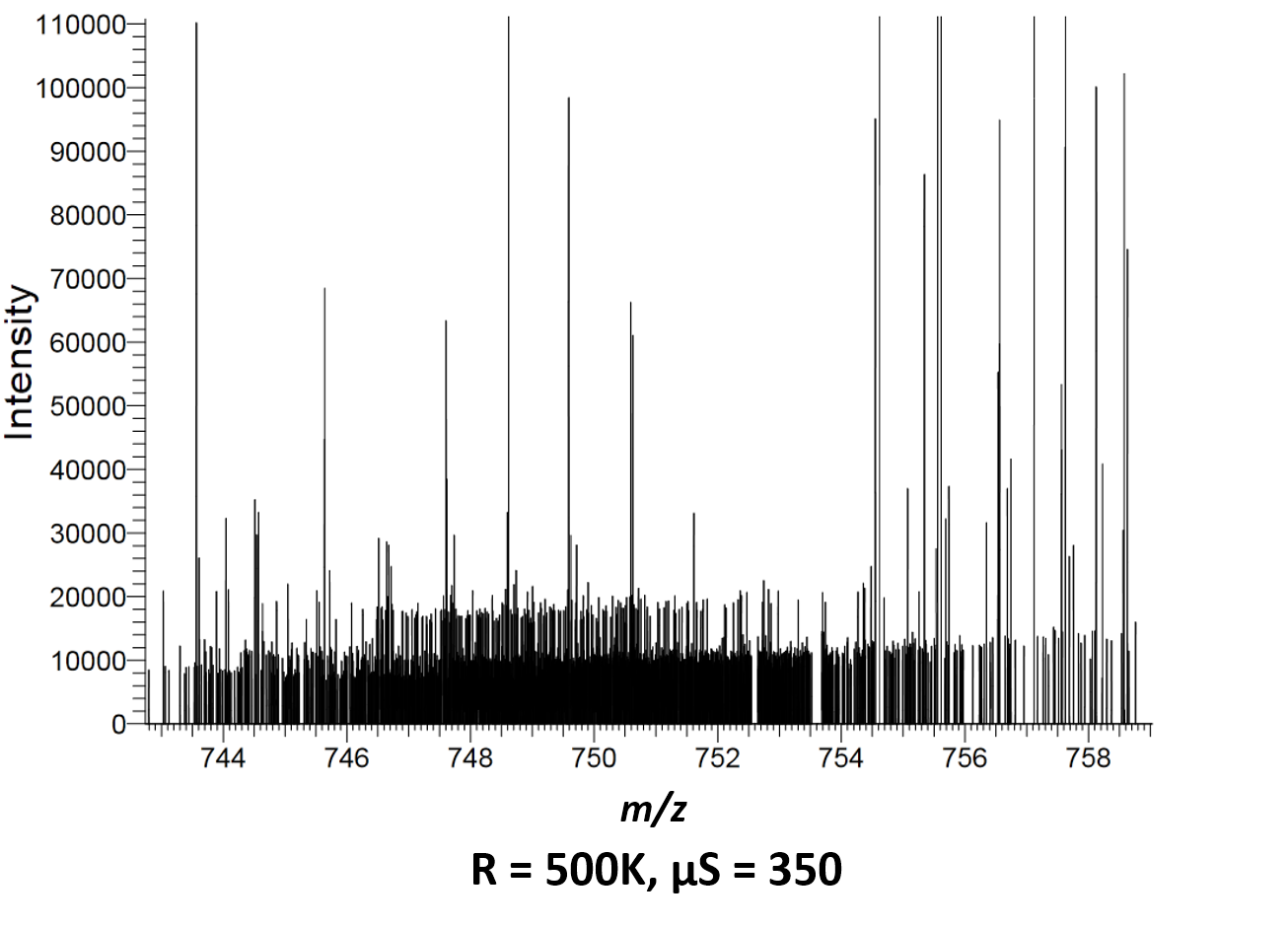


**Figure S-7: Fuzzy Sites vary with Sample Composition.**

A small change in chemical composition (Sample A with and without lipid standards) changes fuzzy site location. With only solvent, there is a fuzzy site at 354.8 *m/z*. With lipid standards, this fuzzy site shifts to 352.1 *m/z*. The number of fuzzy sites will remain constant, but will all be shifted by roughly the same *m/z*. **R** is the resolution setting used for the acquisition, **µS** is the microscan setting, and **N** is the number of scans aggregated to create the spectrum.


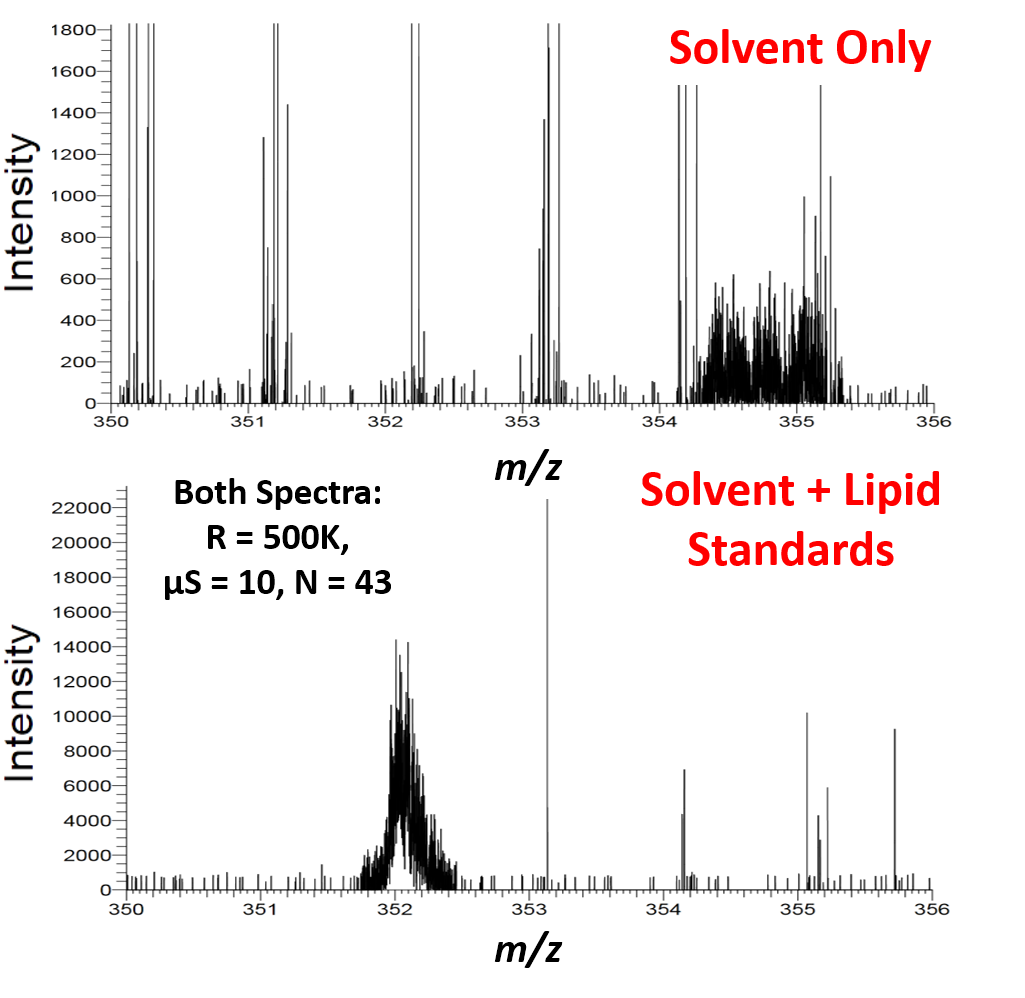


**Figure S-8 Ringing Artifacts**

Ringing results in many artifactual peaks for an intense peak at the scan-level with a distinctive pattern of decaying intensity with increasing distance from the central peak (A). A complete complement of ringing peaks is present for a primary peak in a scan with true ringing. A peak that rings in one scan, does not necessarily ring in other scans; some scans demonstrate partial ringing (C, D) while other scans show no ringing (B). All panels were generated using Sample B.


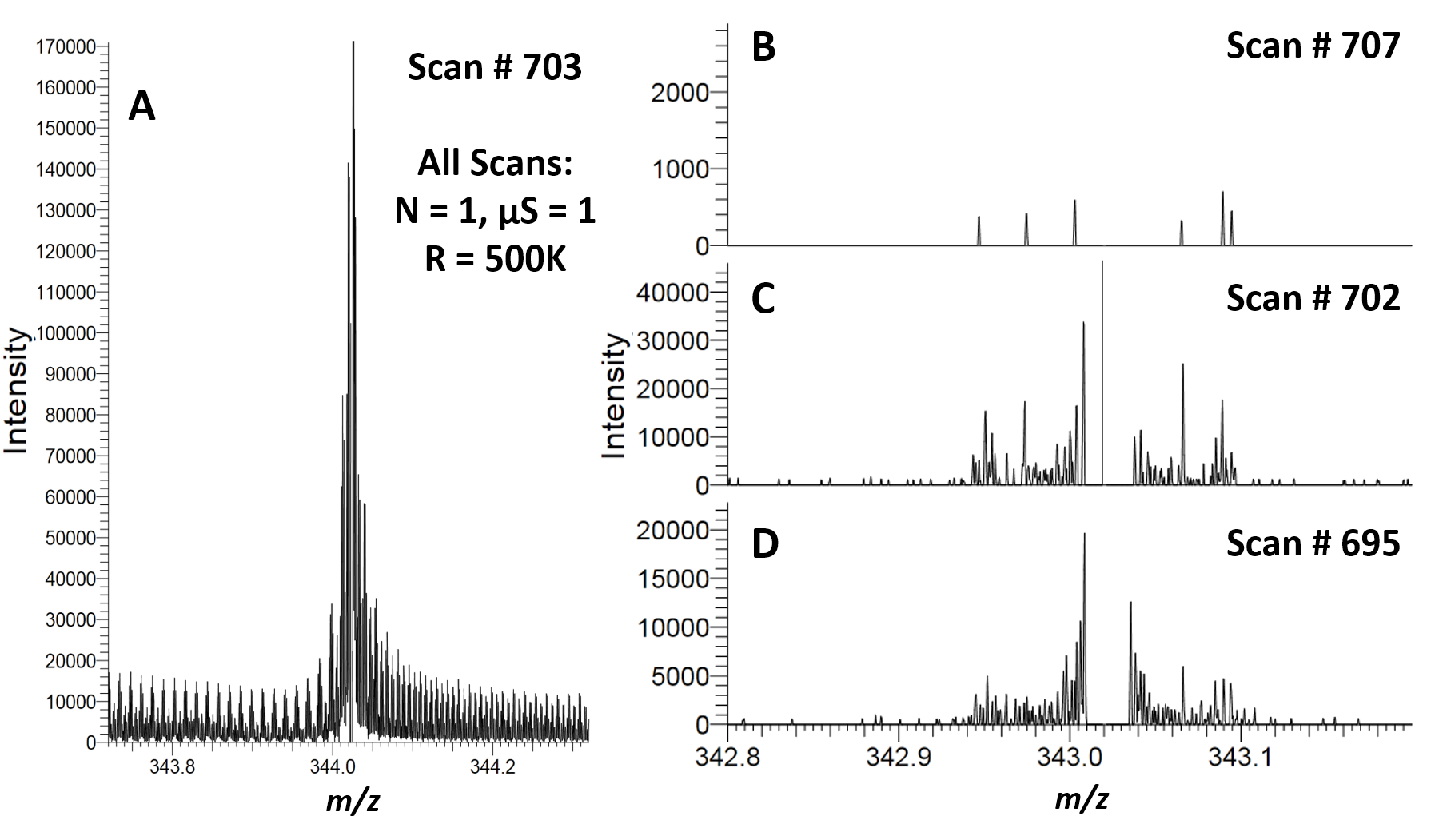


**Figure S-9:**  **Partial Ringing.** (A) Partial ringing produces peak patterns at the aggregate-level that are similar to what ringing produces at the scan-level. Unlike ringing, side peaks of partial ringing do not strictly decrease with increasing distance from the primary peak and are often absent (or greatly diminished) near the primary peak. (B,C,D) At the scan level, the location of the artifactual peaks is highly variable. All panels were generated using Sample C. **R** is the resolution setting used for the acquisition, **µS** is the microscan setting, and **N** is the number of scans aggregated to create the spectrum.


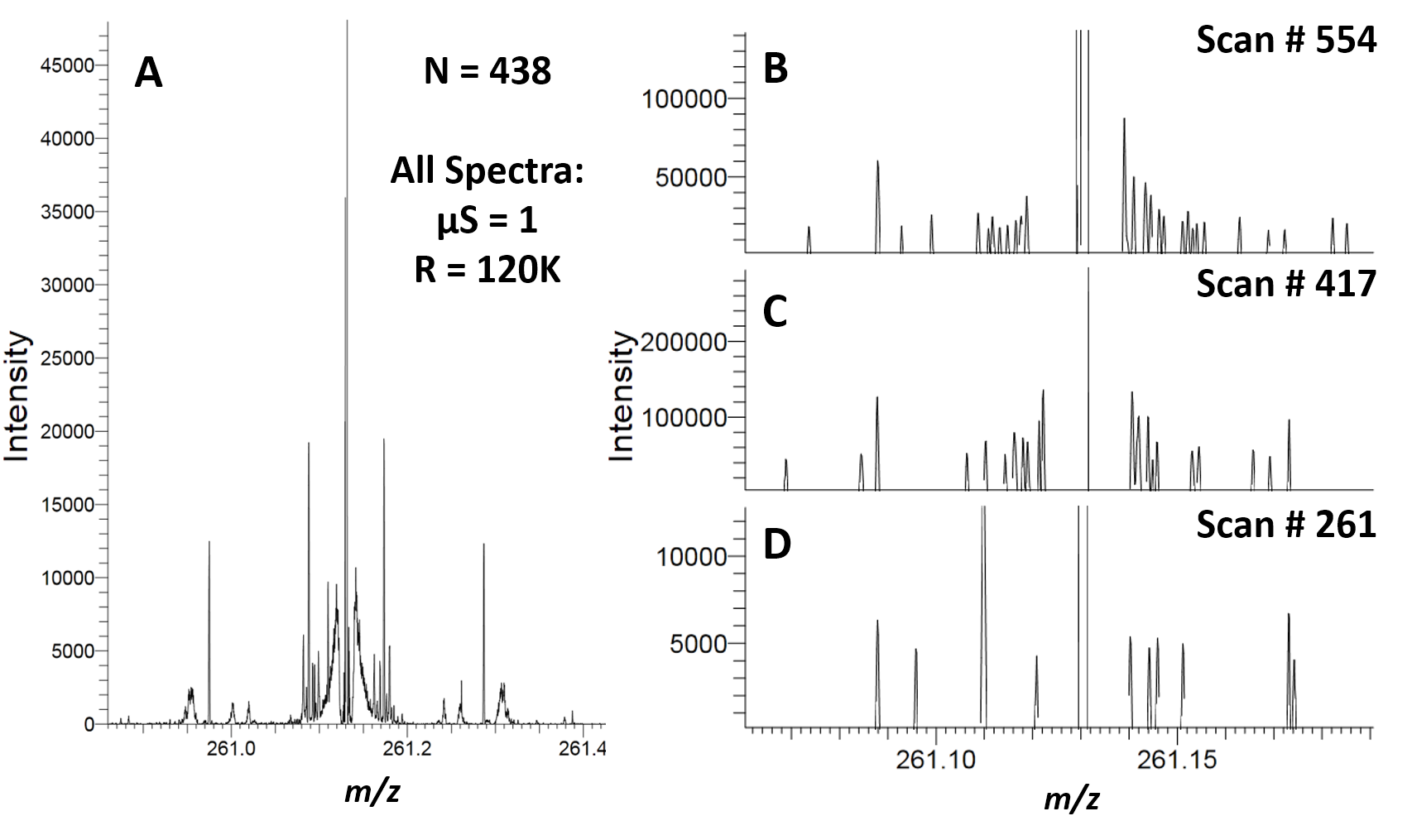


**Supplemental Figure 10**

Not all HPD positive regions contain fuzzy sites; however, the false positive sites do contain abnormal regions of peak density. At the aggregate level, there is no clear fuzzy site, partial ringing or ringing in these examples, but a scan-level analysis shows a characteristic pattern of poor scan-to-scan correspondence for many of the peaks similar to the peaks found in HPD artifacts. For example, Panel A has a scattering of side peaks like filtered partial ringing. Panel B appears to be a thresholded fuzzy site with some non-artifactual peaks as well. Panel C has a few low intensity peaks, but the central peak has a variable width across scans. All spectra acquired using µS =10, R = 500K

**
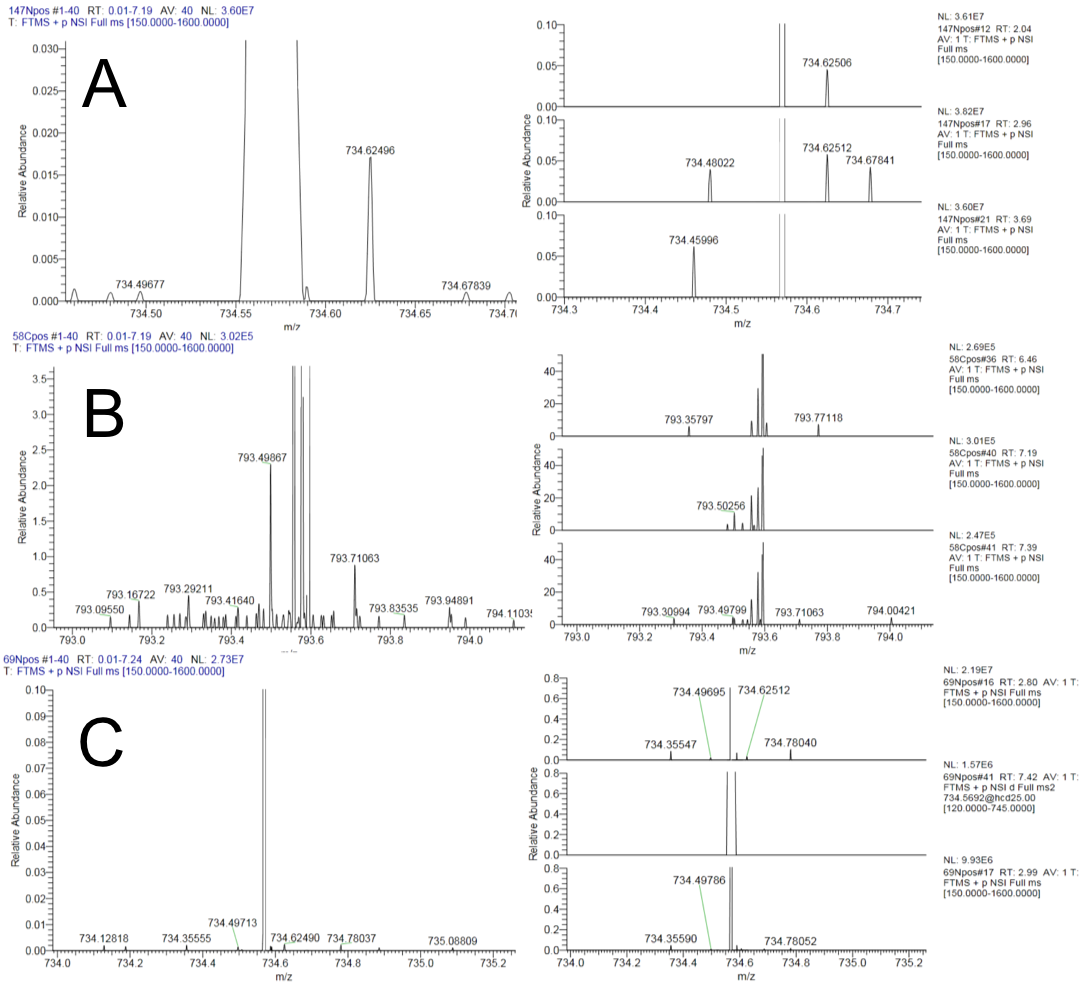
**

**Table S-1: Spectral Acquisition Times for Sample D.**

Spectra for sample D show no significant “batches” with respect to time.Most samples were run sequentially over a time period from 7/2014 to 9/2015. All spectra were acquired using the Fusion 1 and Fusion 2 instruments.

| Sample # | Class | Start Acquisition Time |
| --- | --- | --- |
| 1 | normal | 7/27/2014 10:36 |
| 2 | cancer | 7/27/2014 18:54 |
| 3 | normal | 7/27/2014 19:27 |
| 4 | normal | 7/27/2014 20:35 |
| 5 | cancer | 7/27/2014 21:09 |
| 6 | normal | 7/27/2014 21:43 |
| 7 | cancer | 7/27/2014 22:16 |
| 8 | normal | 7/27/2014 22:50 |
| 9 | cancer | 7/27/2014 23:23 |
| 10 | normal | 7/27/2014 23:57 |
| 11 | cancer | 7/28/2014 0:31 |
| 12 | normal | 7/28/2014 1:05 |
| 13 | cancer | 7/28/2014 1:39 |
| 14 | cancer | 7/28/2014 2:46 |
| 15 | normal | 7/28/2014 3:20 |
| 16 | cancer | 7/28/2014 3:53 |
| 17 | normal | 7/28/2014 4:28 |
| 18 | cancer | 7/28/2014 5:01 |
| 19 | normal | 7/28/2014 5:35 |
| 20 | cancer | 7/28/2014 6:09 |
| 21 | cancer | 7/28/2014 10:39 |
| 22 | normal | 7/28/2014 11:13 |
| 23 | normal | 8/6/2014 13:01 |
| 24 | normal | 8/6/2014 13:46 |
| 25 | normal | 12/19/2014 17:09 |
| 26 | normal | 12/19/2014 18:16 |
| 27 | cancer | 12/19/2014 18:50 |
| 28 | cancer | 12/19/2014 19:58 |
| 29 | normal | 12/19/2014 20:31 |
| 30 | cancer | 12/20/2014 16:26 |
| 31 | cancer | 12/20/2014 17:51 |
| 32 | cancer | 12/20/2014 18:07 |
| 33 | normal | 12/20/2014 18:57 |
| 34 | cancer | 12/20/2014 19:15 |
| 35 | normal | 12/20/2014 19:32 |
| 36 | cancer | 12/20/2014 19:49 |
| 37 | cancer | 1/7/2015 19:38 |
| 38 | normal | 1/7/2015 19:54 |
| 39 | normal | 1/7/2015 21:36 |
| 40 | normal | 1/7/2015 22:09 |
| 41 | normal | 1/7/2015 22:26 |
| 42 | normal | 1/8/2015 12:11 |
| 43 | cancer | 1/8/2015 12:28 |
| 44 | cancer | 1/8/2015 13:19 |
| 45 | cancer | 1/8/2015 13:52 |
| 46 | normal | 1/8/2015 15:37 |
| 47 | normal | 8/18/2015 21:11 |
| 48 | normal | 8/18/2015 21:45 |
| 49 | cancer | 8/18/2015 22:02 |
| 50 | normal | 8/18/2015 22:18 |
| 51 | cancer | 8/18/2015 22:52 |
| 52 | normal | 8/18/2015 23:09 |
| 53 | cancer | 8/18/2015 23:26 |
| 54 | normal | 8/18/2015 23:44 |
| 55 | cancer | 8/19/2015 0:17 |
| 56 | cancer | 8/19/2015 22:49 |
| 57 | normal | 8/19/2015 23:06 |
| 58 | cancer | 8/19/2015 23:22 |
| 59 | normal | 8/19/2015 23:39 |
| 60 | cancer | 8/19/2015 23:56 |
| 61 | normal | 8/20/2015 0:47 |
| 62 | cancer | 8/20/2015 1:04 |
| 63 | normal | 8/20/2015 1:21 |
| 64 | cancer | 8/20/2015 1:38 |
| 65 | normal | 8/20/2015 2:12 |
| 66 | cancer | 8/20/2015 2:28 |
| 67 | normal | 8/20/2015 2:45 |
| 68 | cancer | 8/20/2015 3:02 |
| 69 | normal | 8/20/2015 3:19 |
| 70 | cancer | 8/20/2015 3:36 |
| 71 | normal | 8/20/2015 4:10 |
| 72 | cancer | 8/20/2015 4:27 |
| 73 | normal | 8/20/2015 4:43 |
| 74 | cancer | 8/20/2015 5:00 |
| 75 | normal | 8/20/2015 19:20 |
| 76 | cancer | 8/20/2015 19:37 |
| 77 | normal | 8/20/2015 19:54 |
| 78 | cancer | 8/20/2015 20:10 |
| 79 | normal | 8/20/2015 20:28 |
| 80 | cancer | 8/20/2015 20:44 |
| 81 | normal | 8/20/2015 21:18 |
| 82 | cancer | 8/20/2015 21:34 |
| 83 | normal | 8/20/2015 21:52 |
| 84 | cancer | 8/20/2015 22:08 |
| 85 | normal | 8/20/2015 22:25 |
| 86 | cancer | 8/20/2015 22:42 |
| 87 | normal | 8/20/2015 23:16 |
| 88 | cancer | 8/20/2015 23:33 |
| 89 | normal | 8/20/2015 23:50 |
| 90 | cancer | 8/21/2015 0:06 |
| 91 | normal | 8/21/2015 0:23 |
| 92 | cancer | 8/21/2015 0:41 |
| 93 | normal | 8/21/2015 1:15 |
| 94 | cancer | 8/21/2015 1:31 |
| 95 | normal | 8/21/2015 1:48 |
| 96 | cancer | 8/21/2015 2:05 |
| 97 | normal | 8/21/2015 2:22 |
| 98 | cancer | 8/21/2015 2:38 |
| 99 | normal | 8/21/2015 3:12 |
| 100 | cancer | 8/21/2015 3:30 |
| 101 | normal | 8/21/2015 3:46 |
| 102 | cancer | 8/21/2015 4:03 |
| 103 | normal | 8/21/2015 4:21 |
| 104 | cancer | 8/21/2015 4:37 |
| 105 | normal | 8/21/2015 5:11 |
| 106 | cancer | 8/21/2015 5:28 |
| 107 | normal | 8/22/2015 15:11 |
| 108 | cancer | 8/22/2015 15:28 |
| 109 | normal | 8/22/2015 15:46 |
| 110 | cancer | 8/22/2015 16:03 |
| 111 | normal | 8/22/2015 19:17 |
| 112 | cancer | 8/22/2015 19:34 |
| 113 | cancer | 8/22/2015 20:08 |
| 114 | normal | 9/4/2015 0:14 |
| 115 | cancer | 9/4/2015 0:31 |
| 116 | normal | 9/4/2015 1:05 |
| 117 | cancer | 9/4/2015 1:21 |
| 118 | normal | 9/4/2015 1:39 |
| 119 | cancer | 9/4/2015 1:55 |
| 120 | normal | 9/4/2015 2:12 |
| 121 | cancer | 9/4/2015 2:47 |
| 122 | normal | 9/4/2015 3:04 |
| 123 | cancer | 9/4/2015 3:20 |
| 124 | normal | 9/4/2015 3:37 |
| 125 | cancer | 9/4/2015 3:54 |
| 126 | normal | 9/4/2015 4:27 |
| 127 | cancer | 9/4/2015 4:45 |
| 128 | normal | 9/4/2015 5:02 |
| 129 | cancer | 9/4/2015 5:18 |
| 130 | normal | 9/4/2015 5:36 |
| 131 | cancer | 9/4/2015 6:09 |
| 132 | normal | 9/4/2015 6:26 |
| 133 | cancer | 9/4/2015 6:44 |
| 134 | normal | 9/4/2015 7:00 |
| 135 | cancer | 9/4/2015 7:17 |
| 136 | normal | 9/4/2015 7:50 |
| 137 | cancer | 9/4/2015 8:08 |
| 138 | normal | 9/4/2015 20:14 |
| 139 | cancer | 9/4/2015 20:30 |
| 140 | normal | 9/4/2015 20:48 |
| 141 | cancer | 9/4/2015 21:05 |
| 142 | normal | 9/4/2015 21:21 |
| 143 | cancer | 9/4/2015 21:55 |
| 144 | normal | 9/4/2015 22:12 |
| 145 | cancer | 9/4/2015 22:29 |
| 146 | normal | 9/4/2015 22:46 |
| 147 | cancer | 9/4/2015 23:03 |
| 148 | normal | 9/4/2015 23:36 |
| 149 | cancer | 9/4/2015 23:53 |
| 150 | normal | 9/5/2015 0:10 |
| 151 | cancer | 9/5/2015 0:27 |
| 152 | normal | 9/5/2015 0:44 |
| 153 | cancer | 9/5/2015 1:18 |
| 154 | normal | 9/5/2015 1:34 |
| 155 | cancer | 9/5/2015 1:52 |
| 156 | normal | 9/5/2015 2:08 |
| 157 | cancer | 9/5/2015 2:25 |
| 158 | normal | 9/5/2015 2:59 |
| 159 | cancer | 9/5/2015 3:17 |
| 160 | normal | 9/5/2015 3:33 |
| 161 | cancer | 9/5/2015 3:50 |
| 162 | normal | 9/5/2015 4:07 |
| 163 | cancer | 9/5/2015 4:40 |
| 164 | normal | 9/5/2015 4:58 |
| 165 | cancer | 9/5/2015 5:15 |
| 166 | normal | 9/5/2015 5:31 |
| 167 | cancer | 9/5/2015 5:48 |
| 168 | normal | 9/5/2015 6:22 |
| 169 | cancer | 9/5/2015 6:39 |
| 170 | normal | 9/5/2015 6:56 |
| 171 | cancer | 9/5/2015 7:13 |
| 172 | normal | 9/5/2015 7:30 |
| 173 | cancer | 9/5/2015 8:03 |
| 174 | normal | 9/5/2015 8:20 |
| 175 | cancer | 9/5/2015 8:38 |
| 176 | normal | 9/5/2015 8:54 |
| 177 | cancer | 9/5/2015 9:11 |
| 178 | normal | 9/16/2015 19:22 |
| 179 | cancer | 9/21/2015 20:28 |
| 180 | normal | 9/21/2015 20:45 |
| 181 | normal | 9/23/2015 19:04 |
| 182 | cancer | 9/23/2015 19:22 |
| 183 | normal | 9/23/2015 19:39 |
| 184 | cancer | 9/23/2015 19:56 |
| 185 | cancer | 9/23/2015 20:13 |
| 186 | cancer | 9/23/2015 20:29 |

**Table S-2**

**Detailed analysis of potential fuzzy sites as detected by the fuzzy site detector.**

**FN = False Negative, TN = True Negative, TP = True Positive**

| Fusion 1 | | | | | | | |
| --- | --- | --- | --- | --- | --- | --- | --- |
| Sample | 187 | 351 | 468 | 654 | 976 | 1590 | False Positives |
| 100Cpos | o | TP | TP | TP | TP | TN | 1 |
| 100Npos | FN | TP | TP | TP | TP | TN | 0 |
| 145Npos | FN | TP | TP | TP | TP | TN | 1 |
| 147Cpos | TP | TP | TP | TP | TP | TN | 6 |
| 147Npos | TP | TP | TP | TP | TP | TN | 3 |
| 149Cpos | TP | TP | TP | TP | TP | TN | 4 |
| 149Npos | TP | TP | TP | TP | TP | TN | 3 |
| 150Cpos | FN | TP | TP | TN | TP | TN | 1 |
| 150Npos | FN | TP | TP | TN | TP | TN | 2 |
| 151Cposd | TP | TP | TP | TN | TP | TP | 6 |
| 151Nposc | TP | TP | TP | TN | TP | TN | 2 |
| 154Cposd | TP | TP | TP | TN | TP | TP | 1 |
| 154Npose | TP | TP | TP | TN | TP | TP | 2 |
| 155Cpos | TP | TP | TP | TN | TP | TN | 4 |
| 158Cposd | TP | TP | TP | TN | TP | TP | 1 |
| 158Nposc | TP | TP | TP | TN | TP | TN | 2 |
| 159Cpos | FN | TP | TP | TN | TP | TN | 2 |
| 159Nposc | TP | TP | TP | TN | TP | TN | 1 |
| 160Cpos | FN | TP | TP | TN | TP | TN | 6 |
| 160Nposc | TP | TP | TP | TN | TP | TN | 4 |
| 172Cpos | FN | TP | TP | TN | TP | TN | 3 |
| 172Npos | FN | TP | TP | TN | TP | TN | 4 |
| 174Cpos | TP | TP | TP | TN | TP | TN | 7 |
| 174Npos | FN | TP | TP | TN | TP | TN | 4 |
| 49Cpos | TP | TP | TP | TP | TP | TN | 3 |
| 49Npos | TP | TP | TP | TP | TP | TN | 3 |
| 53Cpos | TP | TP | TP | TP | TP | TN | 2 |
| 53Npos | FN | TP | TP | TP | TP | TN | 2 |
| 58Cpos | TP | TP | TP | TP | TP | TN | 1 |
| 58Npos | FN | TP | TP | TP | TP | TN | 1 |
| 60Cpos | TP | TP | TP | TP | TP | TN | 0 |
| 60Npos | TP | TP | TP | TP | TP | TN | 0 |
| 62Cpos | TP | TP | TP | TP | TP | TN | 0 |
| 62Npos | TP | TP | TP | TP | TP | TN | 2 |
| 63Cpos | TP | TP | TP | TP | TP | TN | 0 |
| 63Npos | FN | TP | TP | TP | TP | TN | 1 |
| 64Cpos | TP | TP | TP | TP | TP | TN | 3 |
| 64Npos | TP | TP | TP | TP | TP | TN | 3 |

| Fusion 2 | | | |
| --- | --- | --- | --- |
| Sample | 1064 | 1275 | False Positive |
| 10bCpos | TN | FN | 1 |
| 10bNpos | TN | TP | 2 |
| 11bCpos | TP | TP | 1 |
| 11bNpos | TP | TP | 1 |
| 12bCpos | TP | TP | 2 |
| 145Cposrr | TN | TP | 6 |
| 155Nposr | TN | TP | 4 |
| 5bCpos | TN | FN | 2 |
| 5bNpos | TP | TP | 1 |

**Table S-3**

**Random Forest importance lists without fuzzy site removal, with encoding removal and consistent fuzzy site region removal.**

Any feature present in a consistent HPD region was marked as an HPD artifact. Twenty random forests, each with 1000 trees were trained using LipidSearch assignments from non-polar extracts prepared from the paired cancer and non-cancer tissue samples from Sample D. Features not present in at least 25% of one class were omitted from classification. Without any artifact removal, on average 7 of the top 30 features are HPD features (A). With simple per-sample artifact removal 3 of the top 30 features are HPD features (B). Both classifiers achieved identical classification performance (D and E). With consistent removal no HPD features are in the importance list and classification accuracy increases slightly (C and F). The consistency of the important non-HPD features across all classifiers indicates that we are removing artifactual information without losing any real information about the biological differences between the classes. All spectra were acquired using Fusion 1.

S-3A – No Artifact Removal

| **No Artifact Removal** | | | |
| --- | --- | --- | --- |
| Rank | Feature | Importance | Consistent HPD |
| 1 | X350 | 1.299373358 | FALSE |
| 2 | X1009 | 0.920384384 | FALSE |
| 3 | X402 | 0.900396072 | FALSE |
| 4 | X349 | 0.820369127 | FALSE |
| 5 | X431 | 0.752355047 | FALSE |
| 6 | X736 | 0.7378473 | FALSE |
| 7 | X742 | 0.725695053 | TRUE |
| 8 | X723 | 0.679898633 | FALSE |
| 9 | X347 | 0.676724331 | FALSE |
| 10 | X401 | 0.658566321 | TRUE |
| 11 | X404 | 0.651728881 | FALSE |
| 12 | X734 | 0.620850897 | FALSE |
| 13 | X726 | 0.607697318 | FALSE |
| 14 | X729 | 0.591199636 | FALSE |
| 15 | X2102 | 0.58378393 | FALSE |
| 16 | X2054 | 0.571041362 | FALSE |
| 17 | X2082 | 0.525453865 | TRUE |
| 18 | X701 | 0.508660104 | FALSE |
| 19 | X743 | 0.500331637 | TRUE |
| 20 | X732 | 0.485208457 | FALSE |
| 21 | X700 | 0.437173853 | FALSE |
| 22 | X162 | 0.390484284 | FALSE |
| 23 | X2085 | 0.372354876 | FALSE |
| 24 | X2056 | 0.370488925 | FALSE |
| 25 | X2055 | 0.366072405 | FALSE |
| 26 | X1506 | 0.365727832 | TRUE |
| 27 | X2083 | 0.365682277 | TRUE |
| 28 | X744 | 0.347132739 | TRUE |
| 29 | X2080 | 0.346694572 | TRUE |
| 30 | X715 | 0.34494085 | FALSE |

S-3B – Per-Spectrum Artifact Removal

| **Per-Spectrum Artifact Removal** | | | |
| --- | --- | --- | --- |
| Rank | Feature | Importance | Consistent HPD |
| 1 | X350 | 1.335764483 | FALSE |
| 2 | X1009 | 0.87809731 | FALSE |
| 3 | X402 | 0.85556647 | FALSE |
| 4 | X349 | 0.842858879 | FALSE |
| 5 | X723 | 0.781824673 | FALSE |
| 6 | X431 | 0.772611039 | FALSE |
| 7 | X347 | 0.761002863 | FALSE |
| 8 | X736 | 0.722306626 | FALSE |
| 9 | X734 | 0.709441924 | FALSE |
| 10 | X726 | 0.681627616 | FALSE |
| 11 | X404 | 0.678943283 | FALSE |
| 12 | X2054 | 0.665312013 | FALSE |
| 13 | X729 | 0.664527344 | FALSE |
| 14 | X2102 | 0.618331145 | FALSE |
| 15 | X701 | 0.528111766 | FALSE |
| 16 | X732 | 0.489612664 | FALSE |
| 17 | X700 | 0.474386984 | FALSE |
| 18 | X2056 | 0.422860516 | FALSE |
| 19 | X162 | 0.413688477 | FALSE |
| 20 | X715 | 0.395241967 | FALSE |
| 21 | X2083 | 0.378109195 | TRUE |
| 22 | X1348 | 0.375178729 | FALSE |
| 23 | X2084 | 0.366118759 | TRUE |
| 24 | X318 | 0.35953324 | FALSE |
| 25 | X2055 | 0.346034991 | FALSE |
| 26 | X373 | 0.337309568 | FALSE |
| 27 | X2085 | 0.330439642 | FALSE |
| 28 | X405 | 0.326077102 | FALSE |
| 29 | X2087 | 0.324229971 | FALSE |
| 30 | X742 | 0.323276991 | TRUE |

S-3C Consistent Artifact Removal

| **Consistent Artifact Removal** | | | |
| --- | --- | --- | --- |
| Rank | Feature | Importance | Consistent HPD |
| 1 | X350 | 1.40349074 | FALSE |
| 2 | X1009 | 0.93821735 | FALSE |
| 3 | X402 | 0.85311723 | FALSE |
| 4 | X431 | 0.84589205 | FALSE |
| 5 | X349 | 0.83529433 | FALSE |
| 6 | X723 | 0.78495987 | FALSE |
| 7 | X736 | 0.74800251 | FALSE |
| 8 | X404 | 0.73411041 | FALSE |
| 9 | X347 | 0.71234883 | FALSE |
| 10 | X734 | 0.70377724 | FALSE |
| 11 | X726 | 0.6889402 | FALSE |
| 12 | X729 | 0.67175299 | FALSE |
| 13 | X2102 | 0.65206486 | FALSE |
| 14 | X2054 | 0.62677106 | FALSE |
| 15 | X701 | 0.5973959 | FALSE |
| 16 | X732 | 0.54356411 | FALSE |
| 17 | X700 | 0.5268463 | FALSE |
| 18 | X2085 | 0.42897734 | FALSE |
| 19 | X2056 | 0.42800707 | FALSE |
| 20 | X162 | 0.42669432 | FALSE |
| 21 | X715 | 0.41585786 | FALSE |
| 22 | X1348 | 0.41221572 | FALSE |
| 23 | X2055 | 0.38577599 | FALSE |
| 24 | X304 | 0.34688235 | FALSE |
| 25 | X318 | 0.34055065 | FALSE |
| 26 | X704 | 0.33858125 | FALSE |
| 27 | X405 | 0.32862873 | FALSE |
| 28 | X2087 | 0.32842285 | FALSE |
| 29 | X373 | 0.32578843 | FALSE |
| 30 | X2015 | 0.32005003 | FALSE |

**S-3 D No Artifact Removal Confusion Matrix**

| No Artifact Removal | | | |
| --- | --- | --- | --- |
|  |  |  |  |
|  |  | True Class | |
|  |  | Cancer | Non-Cancer |
| Predicted | Cancer | 45 | 7 |
|  | Non-Cancer | 2 | 51 |
|  |  |  |  |
|  |  | Class Error | |
|  | Cancer | 0.1346153 | |
|  | Non-Cancer | 0.0377358 | |

**S-3 E Per-Sample Artifact Removal Confusion Matrix**

| Per-Sample Artifact Removal | | | |
| --- | --- | --- | --- |
|  |  |  |  |
|  |  | True Class | |
|  |  | Cancer | Non-Cancer |
| Predicted | Cancer | 45 | 7 |
|  | Non-Cancer | 2 | 51 |
|  |  |  |  |
|  |  | Class Error | |
|  | Cancer | 0.1346153 | |
|  | Non-Cancer | 0.0377358 | |

**S-3 F Consistent Artifact Removal Confusion Matrix**

| Consistent Artifact Removal | | | |
| --- | --- | --- | --- |
|  |  |  |  |
|  |  | True Class | |
|  |  | Cancer | Non-Cancer |
| Predicted | Cancer | 46 | 6 |
|  | Non-Cancer | 2 | 51 |
|  |  |  |  |
|  |  | Class Error | |
|  | Cancer | 0.1153846 | |
|  | Non-Cancer | 0.0377358 | |

**References:**

Acharjee, A., Prentice, P., Acerini, C., Smith, J., Hughes, I.A., Ong, K., Griffin, J.L., Dunger, D. and Koulman, A. (2017) The translation of lipid profiles to nutritional biomarkers in the study of infant metabolism. *Metabolomics* **13,** 25.

Guan, S. and Marshall, A.G. (1997) Linear prediction Cholesky decomposition vs Fourier transform spectral analysis for ion cyclotron resonance mass spectrometry. *Analytical chemistry* **69,** 1156-1162.

Ren, J.-G., Seth, P., Clish, C.B., Lorkiewicz, P.K., Higashi, R.M., Lane, A.N., Fan, T.W.M. and Sukhatme, V.P. (2014) Knockdown of malic enzyme 2 suppresses lung tumor growth, induces differentiation and impacts PI3K/AKT signaling. *Scientific reports* **4**.

Sellers, K., Fox, M.P., Michael Bousamra, II, Slone, S.P., Higashi, R.M., Miller, D.M., Wang, Y., Yan, J., Yuneva, M.O. and Deshpande, R. (2015) Pyruvate carboxylase is critical for non–small-cell lung cancer proliferation. *The Journal of clinical investigation* **125,** 687.

Sun, R.C., Fan, T.W.M., Deng, P., Higashi, R.M., Lane, A.N., Le, A.-T., Scott, T.L., Sun, Q., Warmoes, M.O. and Yang, Y. (2017) Noninvasive liquid diet delivery of stable isotopes into mouse models for deep metabolic network tracing. *Nature Communications* **8,** 1646.

Weber, R.J., Li, E., Bruty, J., He, S. and Viant, M.R. (2012) MaConDa: a publicly accessible mass spectrometry contaminants database. *Bioinformatics* **28,** 2856-2857.

Wood, M.L. and Mark Henkelman, R. (1985) Truncation artifacts in magnetic resonance imaging. *Magnetic resonance in medicine* **2,** 517-526.

Yang, Y., Fan, T.W.M., Lane, A.N. and Higashi, R.M. (2017) Chloroformate derivatization for tracing the fate of Amino acids in cells and tissues by multiple stable isotope resolved metabolomics (mSIRM). *Analytica Chimica Acta* **976,** 63-73.
